# High-throughput phenotyping using digital and hyperspectral imaging-derived biomarkers for genotypic nitrogen response

**DOI:** 10.1093/jxb/eraa143

**Published:** 2020-03-18

**Authors:** Bikram P Banerjee, Sameer Joshi, Emily Thoday-Kennedy, Raj K Pasam, Josquin Tibbits, Matthew Hayden, German Spangenberg, Surya Kant

**Affiliations:** 1 Agriculture Victoria, Grains Innovation Park, Horsham, VIC, Australia; 2 Agriculture Victoria, AgriBio, Centre for AgriBioscience, Bundoora, VIC, Australia; 3 School of Applied Systems Biology, La Trobe University, Bundoora, VIC, Australia; 4 Centre for Agricultural Innovation, School of Agriculture and Food, Faculty of Veterinary and Agricultural Sciences, The University of Melbourne, Victoria, Australia; 5 Michigan State University, USA

**Keywords:** Biomass, chlorophyll, image analysis, nitrogen use efficiency, vegetation indices, wheat

## Abstract

The development of crop varieties with higher nitrogen use efficiency is crucial for sustainable crop production. Combining high-throughput genotyping and phenotyping will expedite the discovery of novel alleles for breeding crop varieties with higher nitrogen use efficiency. Digital and hyperspectral imaging techniques can efficiently evaluate the growth, biophysical, and biochemical performance of plant populations by quantifying canopy reflectance response. Here, these techniques were used to derive automated phenotyping of indicator biomarkers, biomass and chlorophyll levels, corresponding to different nitrogen levels. A detailed description of digital and hyperspectral imaging and the associated challenges and required considerations are provided, with application to delineate the nitrogen response in wheat. Computational approaches for spectrum calibration and rectification, plant area detection, and derivation of vegetation index analysis are presented. We developed a novel vegetation index with higher precision to estimate chlorophyll levels, underpinned by an image-processing algorithm that effectively removed background spectra. Digital shoot biomass and growth parameters were derived, enabling the efficient phenotyping of wheat plants at the vegetative stage, obviating the need for phenotyping until maturity. Overall, our results suggest value in the integration of high-throughput digital and spectral phenomics for rapid screening of large wheat populations for nitrogen response.

## Teaser

A novel vegetation index was derived to estimate chlorophyll and was shown to be effective for high-throughput phenotyping of wheat germplasm for nitrogen response.

## Introduction

Globally, low levels of nitrogen (N) in arable soils limit crop productivity. This constraint has largely been relieved since the development of synthetic N fertilizers, combined with the use of high-yielding crop varieties and irrigation in some parts of the world, all of which are key components of the green revolution. To maximize crop production, large amounts of N fertilizers are applied in regions where farmers can afford to supplement N. Ironically, most crop plants typically utilize only small amounts (<40%) of the applied N (Kant *et al.*, 2011), with the remaining N lost to the environment. This results in higher crop production costs and significant global environmental damage by eutrophication of water ecosystems and gaseous loss to the atmosphere (Gao *et al.*, 2012). To reduce costs and address pollution concerns, improving crop N use efficiency (NUE) by a combination of designing strategies for improved crop management practices, genomics, genetics, and conventional and modern genomic breeding is imperative.

The advent of genomic techniques is transforming plant breeding programmes with rapid genetic gains and shorter breeding cycles. Central to genomic breeding approaches is the availability of high-quality phenotypic data. The development of efficient phenotyping techniques that can rapidly increase throughput, enabling the screening of large numbers of genotypes, is needed to underpin genomic breeding methods. With the increasing availability and decreasing cost of digital sensory image capture and computational analytics, plant phenotyping is in an exponential growth phase. In recent years, advances in computer-vision-assisted sensors and analysis tools have made significant advances in the high-throughput, reliable, and precise phenotyping of plants, as well as for N studies in crops (Fiorani and Schurr, 2013; Deery *et al.*, 2014; Fahlgren *et al.*, 2015; Nguyen and Kant, 2018). The goal of digital imaging is to measure the growth, development and physiology, and to estimate biochemical components of plants, through automated processes. A key component of digital phenotyping is the non-destructive data capture of plant traits, which allows time-series growth measurements, eliminates destructive analysis, and can increase the number of genotypes in an experiment.

Digital sensors and cameras are based on the electromagnetic spectrum and the interaction of plant components with specific spectral regions, such as visible (VIS; 400–700 nm), near infrared (NIR; 700–1000 nm) and shortwave infrared (SWIR; 1000–2500 nm) light (Mulla, 2013; Fahlgren *et al.*, 2015). These digital sensors can be active (with an internal energy source for detecting the response of a crop, e.g. light detection and ranging sensors) or passive [relying on an independent energy source and collecting red-green-blue (RGB) or NIR spectrum light, e.g. multispectral cameras] (Erdle *et al.*, 2011). When light or energy interacts with a plant surface, the radiation is reflected, absorbed, or transmitted. Sensors measure spectral reflectance, and data-processing computational algorithms generate digital plant objects and/or vegetation indices to correlate with plant growth (Li *et al.*, 2001), biomass (Golzarian *et al.*, 2011), physiological status, and biochemical parameters (Homolová *et al.*, 2013; Klukas *et al.*, 2014). Vegetation indices can be identified that correlate with direct phenotyping measurements of traits associated with N, such as chlorophyll levels, protein content, canopy coverage, and biomass (Filella *et al.*, 1995; Erdle *et al.*, 2011; Poiré *et al.*, 2014; Pandey *et al.*, 2017).

Visible-spectrum (RGB) cameras, along with image-processing algorithms, can generate phenotypic traits such as the estimation of biomass, biovolume, leaf area, plant morphology, growth rates, rates of senescence, and pathogen infection (Golzarian *et al.*, 2011; Poiré *et al.*, 2014; Neilson *et al.*, 2015; Cai *et al.*, 2016; Meng *et al.*, 2017; Nguyen *et al.*, 2019). As the wavebands in RGB sensors operate across a relatively broad range of the electromagnetic spectrum, specific wavelength information is lost in the output data. In contrast, hyperspectral sensors can measure hundreds of discrete spectral bands, making hyperspectral imaging a promising technology for the detection and measurement of abiotic stresses (Sytar *et al.*, 2017; Mohd Asaari *et al.*, 2018), biotic stresses (Thomas *et al.*, 2018), and biochemical parameters (Vigneau *et al.*, 2011; Pandey *et al.*, 2017). Image acquisition by hyperspectral sensors can be slower than with other digital sensors, with additional computational challenges in the processing and analysis of high-dimensional hyperspectral data (Bioucas-Dias *et al.*, 2013; Behmann *et al.*, 2016). Nevertheless, plant phenotyping using hyperspectral imaging may be an important component of improving NUE efficiency by enabling the timely, effective, and non-destructive monitoring of N status *in planta*, allowing plant N demand to be better matched with N supply both temporally and spatially (Cassman, 1999; Tilman *et al.*, 2002). Importantly, it would also allow the collection of high-quality phenotypic data in a large diverse germplasm collection such as those used in breeding programmes.

In NUE studies, controlling the plant environment is critical, and genotypes are often initially screened under controlled environmental conditions, with subsets of promising lines selected for further field evaluation. Controlled-environment (e.g. greenhouse, growth chamber) experiments provide greater environmental control, more growing cycles, and are easier environments in which to study root phenotypes, although plants are often constrained by pot dimensions and less natural environmental interactions. For controlled-environment phenotyping, different automated platforms have been described (Li *et al.*, 2014; Poiré *et al.*, 2014; Humplík *et al.*, 2015; Neilson *et al.*, 2015; Pandey *et al.*, 2017), such as the Scanalyzer 3D imaging system, in which plants are placed on a conveyor system and moved to sensors for automated image acquisition (Neilson *et al.*, 2015), PlantEye, a 3D laser scanner mounted on a movable gantry, where a sensor is moved to image the plants (Kjaer and Ottosen, 2015; Vadez *et al.*, 2015; Maphosa *et al.*, 2017), Phenoscope (Tisné *et al.*, 2013), Phenosis (Granier *et al.*, 2006), and Weighing, Imaging and Watering Machines (WIWAM) (Skirycz *et al.*, 2011). 

Wheat is the second most important cereal worldwide after rice (Giraldo *et al.*, 2019). The development of genetic resources and diversity in wheat is well documented and readily available (Hayes *et al.*, 2017; He *et al.*, 2019). Efficient, reliable, objective, and cost-effective phenotyping methods to screen a large wheat germplasm collection under s defined N level will enable these genetic resources to be utilized to improve NUE. Here, we report on the development and testing of efficient protocols with defined varying N levels, and digital and hyperspectral image analysis, to phenotypically screen wheat genotypes under controlled-environment conditions for N response traits. We explored opportunities for phenotyping wheat genotypes in early vegetative growth to obviate the need to grow plants to maturity. Automated phenotyping using visible-colour and hyperspectral cameras, and techniques developed for image processing and analysis, are described to derive biomarkers such as biomass and chlorophyll content, which are important traits for the study of N responses in wheat plants.

## Materials and methods

### Nutrient solution and growth media

Of the two inorganic forms of N (i.e. nitrate and ammonium), nitrate is the predominant N source in most agricultural soils (Kant *et al.*, 2008, 2011). We therefore applied nitrate as the N source for all experiments. Eight N levels were tested, 20, 15, 10, 5, 2, 1, 0.5, and 0.2 mM N, applied as KNO_3_. These N levels were relative, as the nutrient solution containing the indicated N concentration was added once a week. The chosen N regimes were intended to identify optimum N, moderate N stress, and low N stress levels. The other nutrients in the solution were 4 mM MgSO_4_, 4 mM KCl, 5 mM CaCl_2_, 3 mM KH_2_PO_4_/K_2_HPO_4_ (pH 6.0), 0.1 mM Fe-EDTA, 10 µM MnCl_2_, 10 µM ZnSO_4_, 2 µM CuSO_4_, 50 µM H_2_BO_3_, and 0.2 µM Na_2_MoO_4_. A nutrient-free growth medium, consisting of perlite covered with a layer of vermiculite, was used to grow plants. The perlite used was a combination of fine, medium, and coarse grades (2:2:1). As perlite is white and therefore prone to algal growth, which would affect imaging, a layer of vermiculite (~2 cm) was used to cover the surface and prevent algal growth.

### Experiments in a conventional glasshouse

Two separate experiments were conducted. In the first experiment, wheat (*Triticum aestivum* L.) variety Yitpi was grown to full maturity, under the eight N levels described above, with 32 replicates per N treatment, in a semi-hydroponic system using perlite and vermiculite as described above. The second experiment used three N levels, 20, 5, and 2 mM N, which were identified as optimum N, and medium and severe N depletion stress, respectively. The wheat varieties Baxter, Yitpi, Gladius, and Westonia were used in 16 replicates. For both experiments, plastic pots 110×110 mm across and 120 mm deep, with 1 litre capacity, were used; 32 pots, each containing one plant, were placed in a steel tray 50×95 cm and 4 cm deep (see Supplementary Fig. 1A at *JXB* online). Three litres of nutrient solution with a defined N level was applied to each tray at weekly intervals. The growing conditions in the glasshouse were 24 °C/15 °C day/night, with natural lighting. Eight plants per genotype per treatment were destructively harvested at the vegetative growth stage, and the remaining plants were grown to full maturity to obtain yield data.

### Experiments in a high-throughput phenotyping facility

Agriculture Victoria’s Plant Phenomics Victoria, Horsham (PPVH) is a high-throughput controlled-environment phenotyping facility. It is equipped with a conveyor belt system, automated weighing and watering stations, and an automated phenotyping Scanalyzer 3D system (Lemnatec GmBH, Aachen, Germany), which includes high-resolution RGB and hyperspectral imaging sensors. The conveyor belt system comprises 25 housing lanes, each lane accommodating 24 pots, and three separate watering lanes. Each pot is placed in a plastic carrier fitted with a radiofrequency identification (RFID) chip, which is read by RFID chip readers at multiple locations during rotation to ensure accurate pot location during imaging and watering. The RGB imaging system comprises two high-resolution fixed-zoom digital cameras positioned to obtain side and top views. Each RGB camera (Prosilica GT 6600C, Allied Vision Technologies, Stadtroda, Germany) is a 28.8 megapixel camera, with GigE vision compliance and an ethernet interface. Top-view images are acquired from the RGB camera mounted directly above the plant. Side-view images are captured by a rotation function using the ‘turner’, from any angle between 0° and 360°. Depending upon its size, a plant can be lifted using the ‘lifter’ to three levels. The layout of plants on the conveyor belt system and the imaging system are shown in Supplementary Fig. S2.

The hyperspectral camera at PPVH is a pushbroom-type imaging spectrometer (Micro-Hyperspec, VNIR-E Series, Headwall Photonics, Fitchburg, MA, USA) covering extended visible and near-infrared (VNIR) wavelengths. The sensor is operational over a spectral range of 470–1720 nm (the green–red portion of VIS, the entire NIR, and the first part of SWIR) with a spectral resolution of ~4.85 nm, and with a 1:1 binning setting this forms a 256-band hypercube. The sensor is mounted with a fore-optics lenses assembly of 25 mm. The number of horizontal spatial channels (i.e. the number of individual pushbroom detector elements) is 320 pixels wide with an aperture of F/2.5. The position of the hyperspectral sensor from the plant can be adjusted to cover a narrow or wide area depending on the height of the plant. The plant on the conveyor belt system remains stationary during data acquisition and is illuminated with electromagnetic radiation from halogen lamps (Hi-Spot® ES50 Superia, 35W, 180 lumen, beam angle 25°, 2600 K colour temperature) located directly above the plant and on the opposite wall. An internal rotating scanning mirror configuration in the hyperspectral sensor sequentially exposes the detector array with illumination from the imaging chamber in a top-to-bottom direction. The start and stop pitch of the rotating assembly can be programmed to cover both short and tall plants. In this case, the system rotates between 90° (horizontal pitch level) and –110° (inclined pitch level) from the sensor axis at a rate of 0.7° s^–1^, producing a total of 446 scan lines (i.e. 446 vertical pixels). The detector array has a 12-bit radiometric range and transmits the data over ethernet interfaces to a data-acquisition computer.

The PPVH system has three automated weighing and watering stations. Each station has two pumps (Watson-Marlow Fluid Technology Group, Falmouth, UK), one used for watering and other for the application of nutrient solutions. The water or nutrient solution can be dispensed into each pot in multiple ways, such as absolute volume, a target weight, or a target weight with a dynamic offset, depending on the requirement in a given experiment. Weighing scales (Bizerba SE and Co. KG, Balingen, Germany) are used to obtain gravimetric weights before and after watering. To reduce spatial effects, plants were rotated twice a week on the conveyor lanes, such that each whole lane continuously shifted east to west and each half of the lanes rotated north or south.

For the experiments conducted at PPVH, the wheat varieties Baxter, Yitpi, Gladius, and Westonia were grown in two separate experiments with 20, 10, 5, and 2 mM N levels. One plant was grown per pot (200 mm Euro-TL white, Garden City Plastics, Dandenong South, Australia) filled with growth media as described above. Individual pots were weighed and equalized to a fixed pot weight and watered uniformly. Saucers were used to avoid seepage of the nutrient solution. The pots were loaded on to the system 10 days after the emergence of seedlings. Nutrient solution was supplied as 100 ml per pot every week. The growing conditions were 24 °C/15 °C day/night. The two experiments were conducted as biological repeats with 20 replicate plants per N treatment. A subset of five plants per N treatment were destructively harvested at 14, 21, 28 and 35 days after sowing (DAS) and shoot fresh biomass was recorded, and samples were collected for biochemical assays.

### RGB and hyperspectral image acquisition

The RGB images were taken from top and side views (0°, 120°, and 240°), and hyperspectral images were taken from side views. RGB images were analysed using image analysis pipelines developed in the LemnaGrid software (Lemnatec GmBH, Aachen, Germany). An overview of the image analysis pipeline for analysing RGB images is provided in Supplementary Fig. S3. In brief, demosaicing was carried out on raw images to reconstruct a full-colour image, followed by applying a region of interest to separate the plant from the background. The resulting image was converted to the hue-saturation-intensity (HSI) colour space to enhance the separation between plant and background. An appropriate threshold was applied to the image to further improve plant detection. A median filter was used to smooth the edges of the detected image and unwanted solitary pixels were filled by in-painting. Colour classification was applied to delineate the green and non-green tissue and, with the help of image object composition, spatially independent objects were merged into one large object as the plant. The total pixel area was obtained by adding the pixel areas of three side views and the top view, from here on referred to as estimated shoot biomass (ESB). Other morphological parameters estimated included minimum area rectangle (MAR), calliper length (CL), convex hull area (CHA), and eccentricity (E). Details of the measured and estimated morphological parameters are given in Supplementary Table S1 and illustrated in Supplementary Fig. S4.

### Hyperspectral image analysis

Hyperspectral image analysis involved the development of a multi-level processing pipeline for sensor data calibration, illumination adjustment, detection of plant area, and extraction of the plant spectrum, as well as generating standard vegetation indices and applying an innovative brute-force indices mining approach to identify a new vegetation index for the estimation of chlorophyll content.

#### Hyperspectral sensor data calibration

Raw scan lines from the hyperspectral sensor are recorded in digital numbers (DNs) of 12-bit ranges. Spectral and radiometric calibration of the hyperspectral sensor is needed to facilitate transformation of the raw DN values into physical radiance units (mW cm^2^ sr^–1^ µm^–1^) and then to reflectance. Calibration was conducted using a spectralon reference panel (https://www.labsphere.com/) with an optically flat spectral profile at 95% reflectivity, and the sensor operating at 256 spectral bands frame period, with the integration time set at 59 ms. A rectangular section of the spectralon panel (1170 mm length×100 mm width) was placed on the imaging point using a customized height-adjustable mount (Supplementary Fig. 5). The hyperspectral sensor was tuned to focus precisely on the spectralon panel and collect a perfect white spectrum under artificial illumination from the halogen lamps. Additionally, a dark spectrum was collected with the halogen lamps turned off. For each scan line, a corresponding radiometric coefficient—sensor gain and bias transformation were automatically calculated and applied using data-acquisition software (Hyperspec III, Headwall Photonics, Inc) to produce a radiometrically calibrated response. This also removed inter-channel mismatch (flat-field errors) caused by pixel-to-pixel variation in sensitivity in the detector. Automatic hyperspectral image restoration using low-rank and sparse modelling techniques (Rasti *et al.*, 2017) was used to further remove inter-channel variation and radiometric bit error at given pixels.

#### Hyperspectral illumination adjustment

The inside of the hyperspectral imaging cabinet is lined with a reflective material (white in colour) to assist with multiple reflections and scattering, and to produce close-to-uniform illumination conditions. However, the placement of the light source at the top and occlusion produced from the sensor induces a gradient variation in input illumination levels (Supplementary Fig. S6A, B). Moreover, halogen-based lighting systems are prone to brightness changes with heating, drift in radiometric response, and fluctuations due to irregularities in power levels, which affect the standard reflectance response collected by the hyperspectral sensor over time (Supplementary Fig. 6A). To accurately extract physiological traits and chemical properties of plants, hyperspectral imaging relies on the acquisition of systematic reflectance profiles. Some studies have recommended the collection of calibration measurements after each plant measurement (Qin and Lu, 2008), whereas other studies have justified a trade-off between image quality and high throughput (Pandey *et al.*, 2017). We devised a novel illumination-adjustment workflow using persistent radiometric control points to construct a synthetic spatial and temporal profile of illumination errors. A reflectance tarp (56% reflectivity; 163 cm height×117 cm width) was constructed and placed in the background as a calibration panel, so that the tarp remained visible in each hyperspectral image with the plant in the foreground (Supplementary Fig. S5B, C). Four radiometric control points near the corners of the images were selected as references (Supplementary Fig. S6A). A spline function (De Boor, 1978) was used to interpolate an illumination difference or error gradient layer, assuming a 2D Gaussian distribution profile. The illumination error layer was computed for each acquired hypercube and subsequently subtracted from the hypercubes to prepare an illumination-adjusted output (Supplementary Fig. S6B, C) with a standard temporal reflectance response (Supplementary Fig. S6D).

#### Detection of plant area and extraction of plant spectrum

**Fig. 1.**
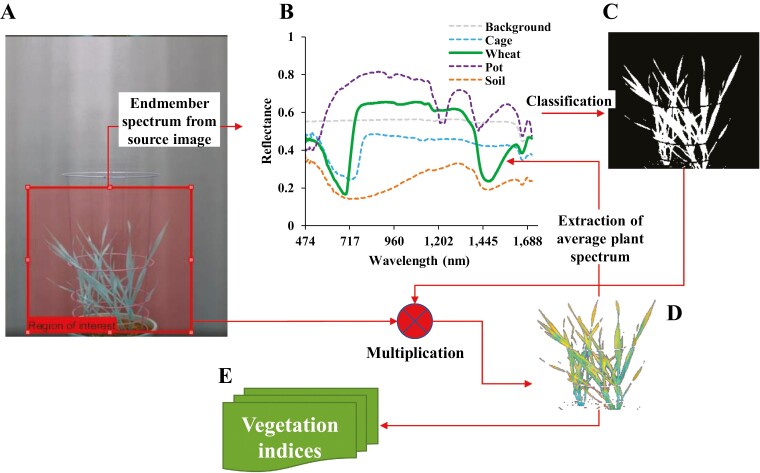
Scheme of image processing and extraction of the plant spectrum. (A) Full plant view with extraction of the region of interest. (B) Extracted endmember spectrum including the wheat plant spectrum. (C) Classified plant region using spectral angle divergence. (D) Extracted wheat plant hypercube. (E) Derived vegetation indices.

A typical hypercube acquisition contains a set of finite image elements—that is, the plant, cage, pot, soil, and background (Fig. 1A). For extraction of vegetation biomarkers and indices, it is essential to first detect the plant pixels within the image. Vigneau *et al.* (2011) have previously used index-based thresholding to separate the plant and the non-plant area. This approach has certain limitations for analysing stressed plants, as tissue tends to change in reflectance and levels of indices usually decrease under stress, meaning that parts of the plant showing stress symptoms are masked off from the analysis due to a stringent index threshold value. We devised a spectrum elimination technique to selectively avoid the inclusion of non-plant (cage, pot, soil, and background) class pixels, thereby detecting both the healthy and the stressed tissue in an imaged plant. The method worked by selecting non-plant-class objects (using image pixels) to create a spectral library, which was used to generate a spectral information divergence layer (Chang, 1999) for the image scene within a spectral divergence angle of 0.2 radians. A binary mask was computed for the pixels remaining (i.e. the plant class) out of the spectral information divergence classification (Fig. 1). A pixel-level multiplication was then performed between the binary mask and the intended hypercube to detect the plant area in the hypercube. For further analysis, the detected plant pixels from the hypercube of an imaged plant were averaged to generate an individual reflectance spectrum with 256 spectral bands.

#### Computation of standard vegetation indices

The generation of vegetation indices from reflectance signatures involved a spectral transformation of reflectance at two or more narrow wavebands designed to highlight a particular property of vegetation. A linear resampling approach based on a generalized Kaiser–Bessel approximation model (Lewitt, 1990) was used to filter and spectrally sample the reflectance signature to the desired narrow wavelengths (1 nm spectral width) as required by the target vegetation indices. After successful resampling, a total of 47 possible standard vegetation indices within the operational spectral range of the hyperspectral system (470–1720 nm) were selected (Supplementary Table S2).

#### Development of a novel vegetation index for estimation of chlorophyll levels

Plants utilize the chlorophyll pigment in leaves to absorb solar energy and convert it into chemical energy. Therefore, the chlorophyll level in a plant is physiologically linked to its yield. The suitability of existing standard vegetation indices relating to chlorophyll levels (chlorophyll *a*, chlorophyll *b*, and total chlorophyll) measured in laboratory destructive analysis was investigated. To further improve the model prediction in the estimation of chlorophyll, a new narrowband index was developed, referred to here as the normalized difference chlorophyll index in wheat (NDCI_W_). Hyperspectral data collected from the first experiment was used to identify suitable wavelengths for NDCI_W_. An indices model was defined using the following function: 

$$\begin{array}{l} indicesmodel=\frac{\rho_{i}-\rho_{j}}{\rho_{i}+\rho_{j}} \end{array}$$ (1)

where *ρ* represents wavelength band over the electromagnetic range with *i* and *j=*470–1720 nm at 1 nm spectral step.

A non-parametric linear regression equation was used as a criterion function to identify suitable band combinations:

$$\begin{array}{l} r=\frac{n(\overset{}{\sum }xy)-(\overset{}{\sum }x)(\overset{}{\sum }y)}{\sqrt{\left[n\overset{}{\sum }x^{2}-{\left(\overset{}{\sum }x\right)}^{2}\right]-[n\overset{}{\sum }y^{2}-{\left(\overset{}{\sum }y\right)}^{2}]}} \end{array}$$ (2)

where *r* is the regression value for the new indices model expressed as *x* and the true value expressed as *y*.

It can intuitively be understood that the optimal bands should permit maximal valuation for |*r*| or *r*^*2*^. The equivalent maximization criterion function is equivalent to maximizing the trace of the correlation as:

$$\begin{array}{l} \underset{\Phi }{argmax}\;\left\{trace[r^{2}]\right\} \end{array}$$ (3)

where Φ is the universal set of all possible paired combination of wavelengths.

**Fig. 2.**
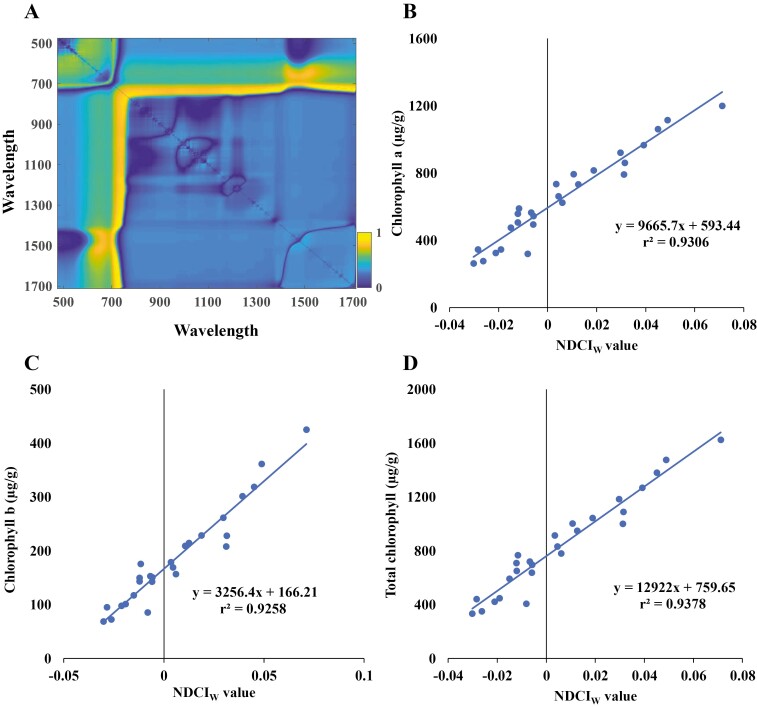
Derivation of the novel vegetation index and correlation with chlorophyll content. (A) Correlation map plot generated using the brute-force indices mining approach in Metlab. (B) Chlorophyll *a*, (C) chlorophyll *b*, and (D) total chlorophyll linear training model correlation with the novel vegetation index, normalized difference chlorophyll index in wheat (NDCI_W_).

Fig. 2A shows the correlation map for all the input combinations of wavelengths expressed on a Cartesian space. The heat map shows the correlation levels, with high levels depicted in bright yellow. It is clear from the plot that there are several potential suitable combinations of wavelength for NDCI_W._ However, the maximal trace was identified for wavebands at 727 nm and 1654 nm. Therefore, NDCI_W_ was expressed as:

$$\begin{array}{l} NDCI_{W}=\frac{\rho_{1654}-\rho_{727}}{\rho_{1654}+\rho_{727}} \end{array}$$ (4)

where, *ρ* represent the reflectance in designated wavelength bands 1654 nm and 727 nm.

The validity of the developed index NDCI_W_ was tested using the dataset from the second experiment.

### Biochemical assays

Leaf tissue was finely ground using a pestle and mortar with liquid N, then aliquoted into 50 mg subsamples and stored at –80 °C until biochemical analysis. Chlorophyll was extracted with 100% methanol followed by centrifugation for 10 min at 10 016 *g*; this process was repeated twice. Extracts were analysed by recording the absorbance at 750, 665, 652, and 470 nm using a UV-VIS spectrophotometer (Shimadzu UV-1800, Shimadzu Inc., Kyoto, Japan). Chlorophyll *a*, chlorophyll *b*, and total chlorophyll were calculated using the formula described in Lichtenthaler (1987).

### Statistical analysis

Linear regression and Pearson’s correlation coefficient (*R*) were used to determine the association between the destructively harvested fresh biomass and digitally estimated plant parameters using the ‘psych’ package in R software (https://cran.r-project.org/web/packages/psych/index.html and http://www.R-project.org).

## Results

### Defining N growth for wheat

**Fig. 3.**
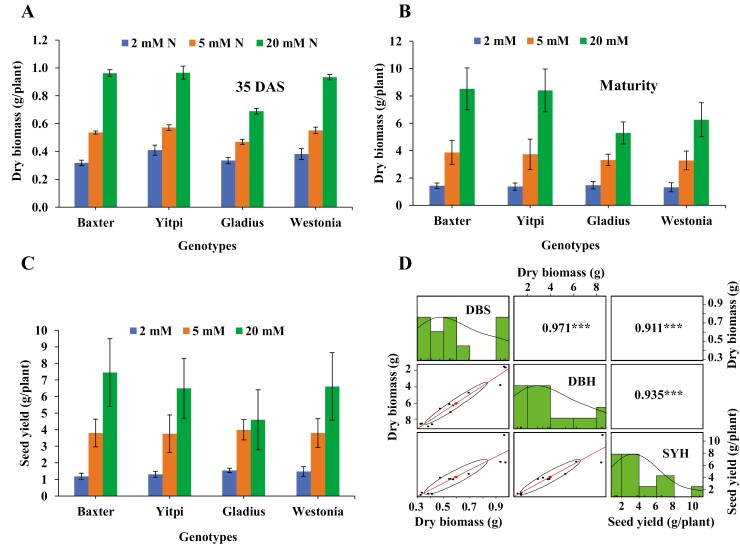
Growth and yield of wheat varieties at different N levels. Dry biomass of four wheat varieties (A) at the vegetative stage, 35 days after sowing (DAS), and (B) at maturity. (C) Seed yield. (D) Correlations between dry biomass at the vegetative and maturity stages and seed yield. DBS, dry biomass at the vegetative stage; DBH, dry biomass at the harvest stage; SYH, seed yield at the harvest stage. The values in the upper right panels indicate pairwise correlation coefficients (*R*). ***P<0.001. The histograms and density plots show the distribution of the data for each trait. The lower left panels show pairwise scatter plots with confidence interval; the ellipse in each plot represents a graphical representation of the extent of relationships; the large dot in the middle of the ellipse indicates the focus of the ellipse; the diagonal line is the line of best fit resulting from linear regression analysis. Data represent the mean ±SEM. (This figure is available in colour at *JXB* online.)

Wheat plants grown in nutrient-free growth medium were evaluated for biomass and yield responses under N levels ranging from 0.2 to 20 mM N (Supplementary Figs S1A and S2C). Dry biomass and yield increased exponentially until 10 mM N, and there was a non-significant increase thereafter (Supplementary Fig. S1B, C). For subsequent experiments, 20 mM N was used as the optimum N condition, 5 mM N as moderate N stress, and 2 mM as low N stress. The four wheat varieties showed an incremental increase in dry biomass and seed yield from low to high N levels. The trend for dry biomass accumulation at both the vegetative growth and the maturity stage as well as seed yield at a given N level were similar across all varieties (Fig. 3A–C). Dry biomass at the vegetative growth stage was highly correlated with dry biomass at maturity and seed yield (Fig. 3D).

### Estimation of shoot biomass and dynamic growth of wheat plants

**Fig. 4.**
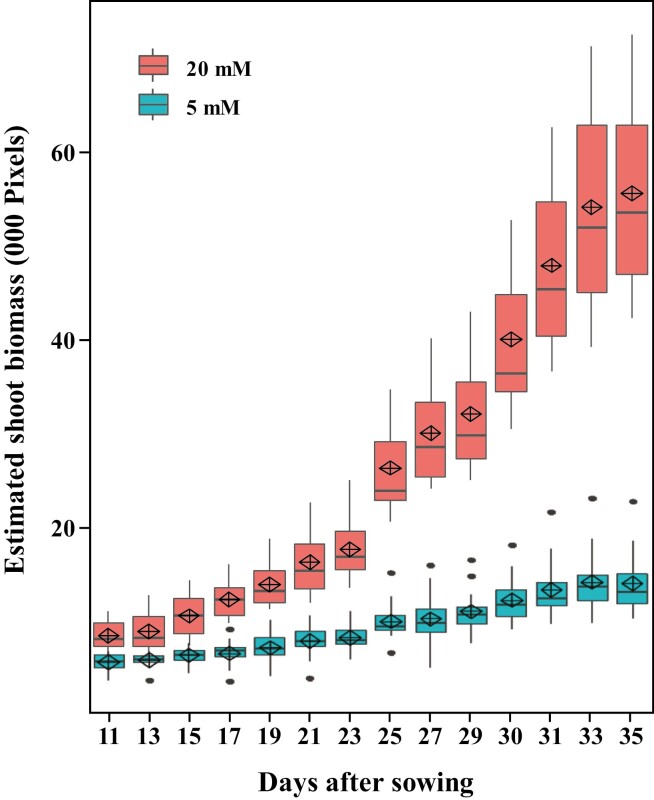
Dynamic growth rate of wheat at varying N levels. Box plot showing the estimated shoot biomass accumulated during the vegetative growth period of plants supplied with 20 and 5 mM N, derived from digital imaging at Plant Phenomics Victoria, Horsham. The black horizontal line represents the median; the diamond indicates the mean; the upper and lower limits of the bounding box represent the 75th and 25th percentiles, respectively; the whiskers extend to the maximum and minimum values excluding outliers, which are plotted individually. (This figure is available in colour at *JXB* online.)

The application of digital sensing techniques for the determination of N response was studied using image-based phenotyping. RGB images were acquired in an automated imaging system at PPVH (Supplementary Fig. S2) and analysed using automated image analysis pipelines (Supplementary Figs S3 and S4) that identify colour-classified wheat plants by removing background elements. ESB was calculated from analysed images by adding together the number of pixels of the three side views and a top view, and was highly correlated with actual shoot fresh biomass (Supplementary Fig. S7A). Images were acquired from early vegetative growth until late reproductive growth. Plants grown in the presence of 20 mM N showed an exponential growth curve in biomass accumulation, while plants supplied with 5 mM N showed a linear increase in biomass during the growing period (Fig. 4 and Supplementary Fig. S7B). The average ESB in 20 mM N-treated plants and 5 mM N-treated plants was 270 and 40 kilopixels, respectively, at 83 DAS (Supplementary Fig. S7B). Importantly, clear differences between the treatments were effectively delineated through digital estimation of shoot biomass at different imaging time points during the early vegetative growth phase (Fig. 4).

The digital parameters MAR, CL, and CHA were also estimated. The 20 mM N-treated plants showed larger MAR, higher CL, and larger CHA compared with 5 mM N-treated plants (Supplementary Fig. S4). The parameter E represents the extent of plant spread. The 5 mM N-treated plants had higher E values than the 20 mM N-treated plants, with mean values of 0.74 and 0.92, respectively (data not shown). The relationship between measured and estimated parameters was determined by Pearson’s correlation analysis. A very high positive correlation was observed between harvested fresh biomass and ESB (*R*=0.986), MAR (*R*=0.921), CL (*R*=0.898), and CHA (*R*=0.929), while a very high negative correlation was recorded for E (*R*=–0.883) (Supplementary Fig. S8).

### Correlation of the novel vegetation index NDCI_W_ with measured chlorophyll levels in wheat

The performance of the novel vegetation index, NDCI_W_, and 47 published indices were tested for chlorophyll estimation (Supplementary Tables S2 and S3). To describe the ability of the previously published spectral indices to measure chlorophyll levels as a means of assessing N treatments, we used the datasets from two experiments to calculate the Pearson correlation (*r*^2^) relationship between the indices and values of total chlorophyll level measured by laboratory-based biochemical assays (Supplementary Table S3). Most published vegetation indices demonstrated a low correlation with measured chlorophyll levels (*r*^2^<0.5). Some indices, such as the Vogelmann Red Edge Indices 1, 2, and 3 (VOG1, VOG2, and VOG3), the Transformed Chlorophyll Absorption Reflectance Index (TCARI), and the Zarco-Tejada and Miller Index (ZMI), achieved a reasonable degree of correlation with chlorophyll levels, although the correlation was not consistent across two experiments and/or did not work consistently for chlorophyll *a*, chlorophyll *b*, and/or total chlorophyll. For instance, VOG1 had correlations with chlorophyll *a* (*r*^2^=0.65), chlorophyll *b* (*r*^2^=0.69), and total chlorophyll (*r*^2^=0.66) in experiment 1, but in experiment 2 these correlations were *r*^2^=0.80, *r*^2^=0.48, and *r*^2^=0.76, respectively.

**Fig. 5.**
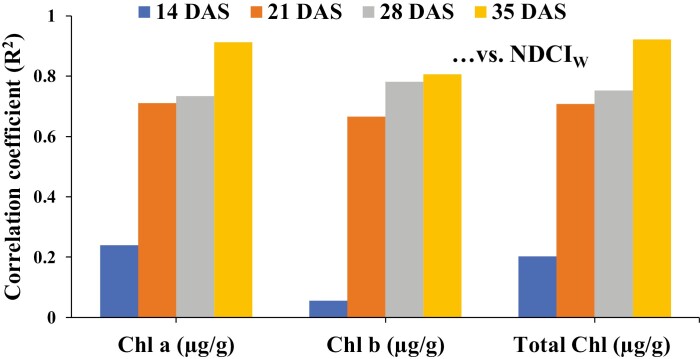
Correlation of chlorophyll (Chl) *a*, Chl *b*, and total Chl with the novel vegetation index, NDCI_W_, at different growth stages. DAS, days after sowing. (This figure is available in colour at *JXB* online.)

The new vegetation index (NDCI_W_) achieved consistently high correlations with chlorophyll *a* (*r*^2^=0.93), chlorophyll *b* (*r*^2^=0.93), and total chlorophyll (*r*^2^=0.94), and outperformed the other standard indices (Supplementary Table S3). To validate the potential of NDCI_W_ to retrieve information on chlorophyll levels, the transferability of the index was tested on a separate experimental dataset. While the correlation with measured chlorophyll levels was low at a very early stage of plant development (14 DAS), due to limited numbers of available plant pixels in very small plants, the correlation rapidly increased from 21 DAS onwards (Fig. 5). The accuracy of NDCI_W_ in predicting chlorophyll saturated between 21 DAS and 35 DAS, achieving the highest correlations at 35 DAS with chlorophyll *a* (*r*^2^=0.91), chlorophyll *b* (*r*^2^=0.81), and total chlorophyll (*r*^2^=0.92) (Fig. 5).

**Fig. 6.**
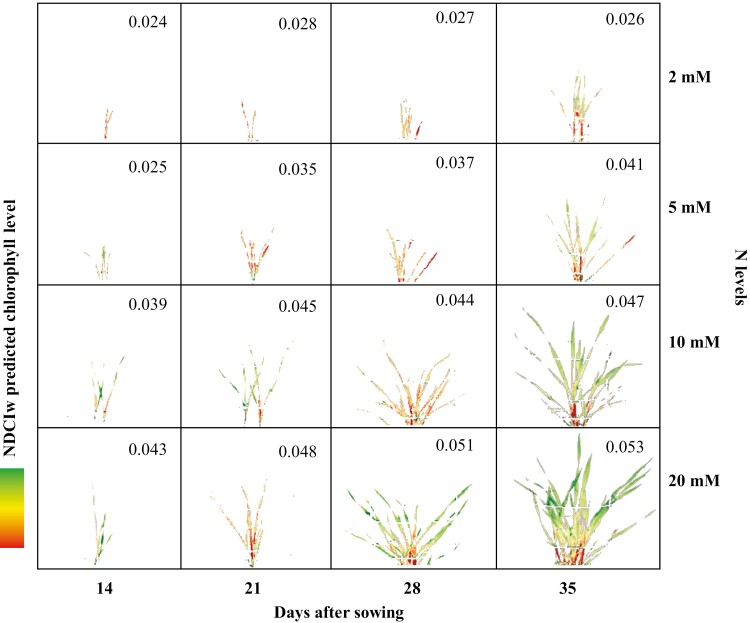
Chlorophyll estimation and growth profile of wheat plants at different N levels. False-colour composite (red-yellow-green) generated from hyperspectral data showing NDCI_W_ profile as an indicator of chlorophyll at different imaging time points (x axis) and N levels (y axis). The numbers in the panels are average NDCIw values for all experimental plants at the corresponding imaging time and N level.

A false-colour scheme (red-yellow-green in increasing order of response) was used to represent the chlorophyll levels predicted in representative plants from experiment 2 using the NDCI_W_ (Fig. 6). At the first time point (14 DAS), plants at all N treatment levels have similar plant area and predicted chlorophyll levels, although the 10 mM and 20 mM N-treated plants on average contained more chlorophyll. At later imaging time points (21 DAS onwards) significant N treatment responses were seen, with 20 mM N-treated plants demonstrating the highest estimated chlorophyll levels compared with the other N levels (Fig. 6). Fig. 6 also shows the average predicted chlorophyll levels for all imaged plant biomass across all biological replicates within a treatment. Chlorophyll levels for plants treated with 2 mM N did not change across the growth period because, even though the biomass increases, leaves cannot be kept alive. Although 5 mM N-treated plants showed an increase in chlorophyll level, the increase was gradual and levels remained low. Plants grown with 10 mM and 20 mM N started with high predicted chlorophyll levels at 14 DAS, which increased with biomass production (Fig. 6). These trends in predicted chlorophyll levels closely matched the actual chlorophyll concentrations measured in plants harvested on the same imaging days (Supplementary Fig. S9). Clear treatment differences between the four N treatments, were evident from 21 DAS onwards for chlorophyll *a*, chlorophyll *b*, and total chlorophyll (Supplementary Fig. S9). The close match between measured chlorophyll concentrations and predicted chlorophyll levels provides further evidence of the strength of the NDCI_W_ in the non-destructive estimation of chlorophyll levels. The natural concentration gradient of chlorophyll, with high levels in young (upper) leaves and lower levels in older (lower) leaves, was particularly visible at 35 DAS (Fig. 6). The gradient was less prominent in the low-N treatments (2 nM and 5 nM), with the colour scheme being centred near low levels of NDCI_W_. These false-colour images generated using NDCI_W_ data could also be used to track the location of and changes in photosynthetically active tissues, especially in N stressed plants, where symptoms may not develop clearly in the visible light spectrum.

## Discussion

Increasing NUE in crop species will play an important role not only in improving plant growth, development, and yield, but also in helping to reduce excess N contamination of the atmosphere, soil, and ground water (Good and Beatty, 2011; Cormier *et al.*, 2013). Modern genomic breeding strategies rely on efficient genotyping and phenotyping for NUE breeding, that is, by studying the phenotypic N responses in diverse wheat germplasm. Conventional phenotyping for N response relies on manual and/or destructive analysis of biomarkers such as plant growth (mainly biomass), biochemical components (chlorophyll, protein, and N content), and grain yield, which increases the cost and time required (Furbank and Tester, 2011). Technologies that are more cost and time efficient than traditional phenotyping methods are therefore key to crop breeding programmes (Yendrek *et al.*, 2017; Silva-Perez *et al.*, 2018). 

In this study, protocols were developed with defined varying N levels, and an image-based, automated, non-destructive phenotyping workflow was established to study N responses in wheat genotypes. We report a simplified system to delineate wheat plant growth responses when grown under varying N levels, which allows the efficient and non-destructive phenotyping of large germplasm collections. Non-destructive and repetitive phenotyping for traits of interest will help in the selection of genotypes without compromising the accuracy of conventional phenotyping methods (Jiménez *et al.*, 2017). Digital and manual observations were recorded at multiple time points throughout the life cycle of wheat plants. Furthermore, repeated digital measurements allowed the measurement of N responses at various growth stages and also the study of dynamic growth curves across time points, which can assess plant N responses with greater precision than manual observations. Our results indicated that N responses during the vegetative stages are highly correlated with N responses in later growth stages in the accessions screened (Fig. 3). Previous research has also shown that screening in the early vegetative stages can provide key insights into plant behaviour and yield during later growth (Kirda *et al.*, 1992; Damon and Rengel, 2007; Krishnamurthy *et al.*, 2007; Nakhforoosh *et al.*, 2016; Meng *et al.*, 2017; Nguyen *et al.*, 2019).

To identify a high-throughput methodology to phenotype wheat for N response, image-based phenotyping using RGB and hyperspectral imaging was conducted at the PPVH facility. RGB images were acquired using a Lemnatec 3D Scanalyzer and plant biomarkers such as ESB, MAR, CL, CHA, and E were found to correlate well with fresh biomass. Similarly high correlations between actual and estimated shoot dry biomass were observed by Golzarian *et al.* (2011) in different wheat varieties. Clear differences in growth, in terms of estimated plant parameters, were observed between high- and low-N-treated plants, which demonstrated the ability of image-based phenotyping to capture both large and subtle variations between treatments. There were high correlations between measured biomass and estimated plant traits at 35 DAS (Supplementary Fig. S8), as well as high correlations between dry biomass at the vegetative stage and both dry biomass at maturity and seed yield (Fig. 3D). This finding suggests that wheat can be effectively screened at the vegetative stage, removing the need to grow plants to maturity, thus increasing throughput and allowing larger populations to be screened in a given timeframe.

Hyperspectral sensors are highly sensitive systems that can be used to measure minute variations in plant reflectance responses when subjected to stress conditions. This high sensitivity also makes a hyperspectral sensor prone to radiometric abnormalities due to spectral or illumination variations. The necessity of proper spectral and radiometric calibration when studying plants in controlled imaging cabinets was previously outlined by Pandey *et al.* (2017). The current study identified similar challenges in calibrating the hyperspectral sensor to accurately measure N responses in wheat. In addition, fluctuations in the intensity of illumination were found to severely affect the detection of plant area across multiple time point images and masked the reflectance response to the applied N treatment variations. The novel illumination adjustment protocol for hyperspectral imaging proposed in this study to stabilize the response due to changing illumination levels allowed a more accurate detection of plant area and profiling of the reflectance response to N levels.

The orientation of the plant with respect to the source of illumination and the hyperspectral sensor places additional constraints on the correct estimation of chlorophyll content. Vegetation indices show anisotropy depending on the structural development of the canopy, shadowing, the view angles of the sensors, the inherent viewing geometry of sensors, and in some respects the underlying soil (Kimes *et al.*, 1985). Vigneau *et al.* (2011) used Lambertian transformation of reflectance to normalize the angular spectral response in wheat. A multi-angular imaging approach was also used by Guo *et al.* (2018) for estimating N uptake using hyperspectral imaging in winter wheat. In the current study, the improved chlorophyll estimation achieved using the new NDCI_W_ index is due to (i) using multi-angular imaging with three views each at 120° offset, then averaging spectral responses; (ii) collecting hyperspectral data at a high sensor zenith angle (~90°) through side-view imaging, thus including total wheat plant characteristics in the lower, middle, and upper canopy; and (iii) using the extended VNIR wavelengths, discussed below.

Chlorophyll plays an important role in photosynthesis and hence can be a direct indicator of a plant’s primary production and photosynthetic potential (Richardson *et al.*, 2002). Chlorophyll content, either measured or estimated, can also be used to determine the N status and stress response of crop plants (Wood *et al.*, 1993; Filella *et al.*, 1995), with remote and non-destructive estimation of chlorophyll content and fluorescence now becoming common (Richardson *et al.*, 2002; Murchie and Lawson, 2013). Previous scientific efforts to non-destructively measure the chlorophyll content of leaves have focused more on the VNIR (400–1000 nm) portion of the electromagnetic spectrum. As part of developing the hyperspectral image analysis pipeline, we tested 47 previously published vegetation indices (between 470 and 1720 nm) related to the biophysical and biochemical basis of plants, many of which make use of the inverse relationship between red and NIR reflectance associated with healthy green vegetation. Different vegetation indices, such as the chlorophyll index (CI), Merris terrestrial chlorophyll index (MTCI), modified chlorophyll absorption in reflectance index (MCARI), and normalized difference red-edge index (NDRE), have been found to indirectly infer the amount of chlorophyll by measuring variation in wavelengths over the NIR, red-edge (RE), and red bands. The superior performance of NDCI_W_ was to a certain extent due to the inclusion of input wavelengths in index computation from near the RE (727 nm) and the extended VNIR (1654 nm) regions. However, the inclusion of widely separated wavelength bands (727 and 1654 nm) also ensured that the output response for NDCI_W_ is high for ‘soil’; that is, the response is sensitive to the presence of exposed growth media. Previous studies have identified the soil effect, or soil brightness as noise in vegetation indices (Richardson and Wiegand, 1977; Major *et al.*, 1990). The effect of soil brightness could be normalized either by tuning the selection of the wavelengths, where possible, or by employing a vegetation masking criterion to avoid the inclusion of soil in the computation of vegetation indices. The latter approach was adopted in this study to mask out the vegetation portion of wheat from the hyperspectral data using a robust calibration and classification workflow before computing the vegetation index, thereby eliminating the soil effect. Additionally, the side-wise imaging geometry helped to reduce the soil brightness effect, as the soil part is limited to the bottom of the image, and the background was a standard reflectance calibration target. 

As shown in this study, the ability to measure the extended VNIR region in controlled environments provides increased capability to detect variations in the concentration of photosynthetically active chlorophyll pigments. The performance of the newly developed NDCI_W_ index was better than that of other available vegetation indices previously proposed for chlorophyll measurement. The employed brute-force indices mining approach utilized a simplistic model with selection of two model input parameters, that is, reflectance at specific wavebands. Furthermore, the indices mining approach developed could be used to extract other physiochemical parameters important for crop growth. Although this research utilized a brute-force indices mining approach to identify a novel index, there are several other noteworthy approaches, including neural networks, machine learning, and deep learning, for chlorophyll estimation. However, these approaches would process the entire spectrum instead of utilizing specific reflectance wavelengths for deriving plant chlorophyll levels. An evaluation of these potential approaches is an important scientific question and requires future research.

Spectral reflectance has previously been shown to be significantly affected by canopy structure, planting density, and the angle of the incident radiation (Rondeaux *et al.*, 1996). Accurate profiling of chlorophyll levels using a vegetation index as an indicator depends on the surface area of the leaves available for spectral imaging. The growth stage of a wheat plant governs the amount of leaf tissue available for imaging. Plants in vegetative growth stages (between 21 and 35 DAS) are therefore the optimum size for hyperspectral imaging, as they have enough pixel area to decipher genotypic and treatment variations but are not too big to cause oversaturation, and also minimize leaf overlap and shadowing. Early vegetative stage plants (21 DAS onwards) showed clear treatment differences in biomass as well as estimated chlorophyll, with estimated chlorophyll levels able to be used to map changes in photosynthetically active tissue as plants develop. Vegetative screening using digital imaging and the new NDCI_W_ index to estimate chlorophyll content should therefore be a successful strategy for high-throughput NUE phenotyping of large wheat populations. The success of NDCI_W_ in wheat also suggests its application to other plants, or at least cereal crops. In the absence of more experimental data on a diverse variety of species, this new index has been catalogued as NDCI_W_ (a novel chlorophyll index in wheat). The expected adoption of this index in future studies by the wider scientific community would potentially aid the evaluation of NDCI_W_ for its relevance to other plants or crop varieties.

In conclusion, spectroscopic methods are useful to estimate growth and biochemical biomarkers of a crop germplasm population by quantifying canopy reflectance across a range of narrowband wavelength channels. In this study, digital and hyperspectral imaging techniques were used to isolate specific phenotypic responses to different N levels and to non-destructively quantify biomass, growth rate, and chlorophyll levels in wheat genotypes grown in the presence of different N levels. Hyperspectral imagery was used to derive a novel vegetation index, NDCI_W_, for the estimation of chlorophyll, which had significantly greater reliability than previously published vegetation indices. An image-processing algorithm was developed that selectively targeted wheat spectra and removed background spectra from hyperspectral sensor data. Furthermore, shoot biomass was estimated using digital coloured imagery to draw dynamic growth curves, which were highly correlated with biomass accumulation during the later stages of growth. The results of both digital and hyperspectral imaging suggest that biomass and chlorophyll estimation can be used as biomarkers to phenotype wheat plants for N response at vegetative stages, obviating the need to phenotype plants up to maturity. A combination of hyperspectral chlorophyll and digital biomass estimations could be used in further studies to non-destructively and rapidly phenotype large wheat populations in a short timeframe, thus speeding up the selection of elite germplasm for NUE breeding programmes.

## Supplementary data

Supplementary data are available at *JXB* online.

Fig. S1. Growth of wheat plants at different nitrogen levels.

Fig. S2. Wheat plants growing in the Plant Phenomics Victoria, Horsham, automated glasshouse.

Fig. S3. Simplified RGB colour image analysis pipeline.

Fig. S4. Estimation of plant digital parameters.

Fig. S5. Hyperspectral sensor system assembly.

Fig. S6. Calibration of illumination variation in hyperspectral sensing.

Fig. S7. Estimation of shoot biomass and growth rate of wheat plants.

Fig. S8. Correlations between estimated and measured plant parameters.

Fig. S9. Chlorophyll analysis of wheat plants.

Table S1. Measured and estimated plant traits using destructive harvesting and digital imaging methods.

Table S2. Computation of published vegetation indices.

Table S3. Correlation analysis of standard vegetation indices and NDCI_w_ for chlorophyll estimation.

eraa143_suppl_Supplementary_Figure

eraa143_suppl_Supplementary_Table
