# Corrigendum to: High-throughput phenotyping using digital and hyperspectral imaging-derived biomarkers for genotypic nitrogen response

**DOI:** 10.1093/jxb/erab126

**Published:** 2021-05-24

**Authors:** Bikram P Banerjee, Sameer Joshi, Emily Thoday-Kennedy, Raj K Pasam, Josquin Tibbits, Matthew Hayden, German Spangenberg, Surya Kant

**Affiliations:** 1 Agriculture Victoria, Grains Innovation Park, Horsham, VIC, Australia; 2 Agriculture Victoria, AgriBio, Centre for AgriBioscience, Bundoora, VIC, Australia; 3 School of Applied Systems Biology, La Trobe University, Bundoora, VIC, Australia; 4 Centre for Agricultural Innovation, School of Agriculture and Food, Faculty of Veterinary and Agricultural Sciences, The University of Melbourne, Victoria, Australia

Journal of Experimental Botany, Volume 71, Issue 15, 25 July 2020, Pages 4604–4615, https://doi.org/10.1093/jbx/eraa143

The original version of this article inadvertently incorporated a typo error in the proposed vegetation index (VI), i.e. normalized difference chlorophyll index in wheat (NDCI_W_) equation 4 on page 4608. The wavelength numbers in the proposed index got assigned in a reversed order than it was intended. The incorrect equation is:


$$\begin{array}{l} NDCI_{W}=\frac{\rho_{727}-\rho_{1654}}{\rho_{727}+\rho_{1654}} \end{array}$$

This equation should be replaced by the correct equation and a following sentence as below:


$$\begin{array}{l} NDCI_{W}=\frac{\rho_{1654}-\rho_{727}}{\rho_{1654}+\rho_{727}} \end{array}$$

where, *ρ* represent the reflectance in designated wavelength bands 1654 nm and 727 nm.

