# Supplementary material for: High-throughput phenotyping using digital and hyperspectral imaging-derived biomarkers for genotypic nitrogen response
Source: J Exp Bot. 2020 Mar 18;71(15):4604–15. doi: 10.1093/jxb/eraa143 (PMC7382386; doi:10.1093/jxb/eraa143)
Supplement: eraa143_suppl_Supplementary_Figure [file eraa143_suppl_supplementary_figure.pdf]

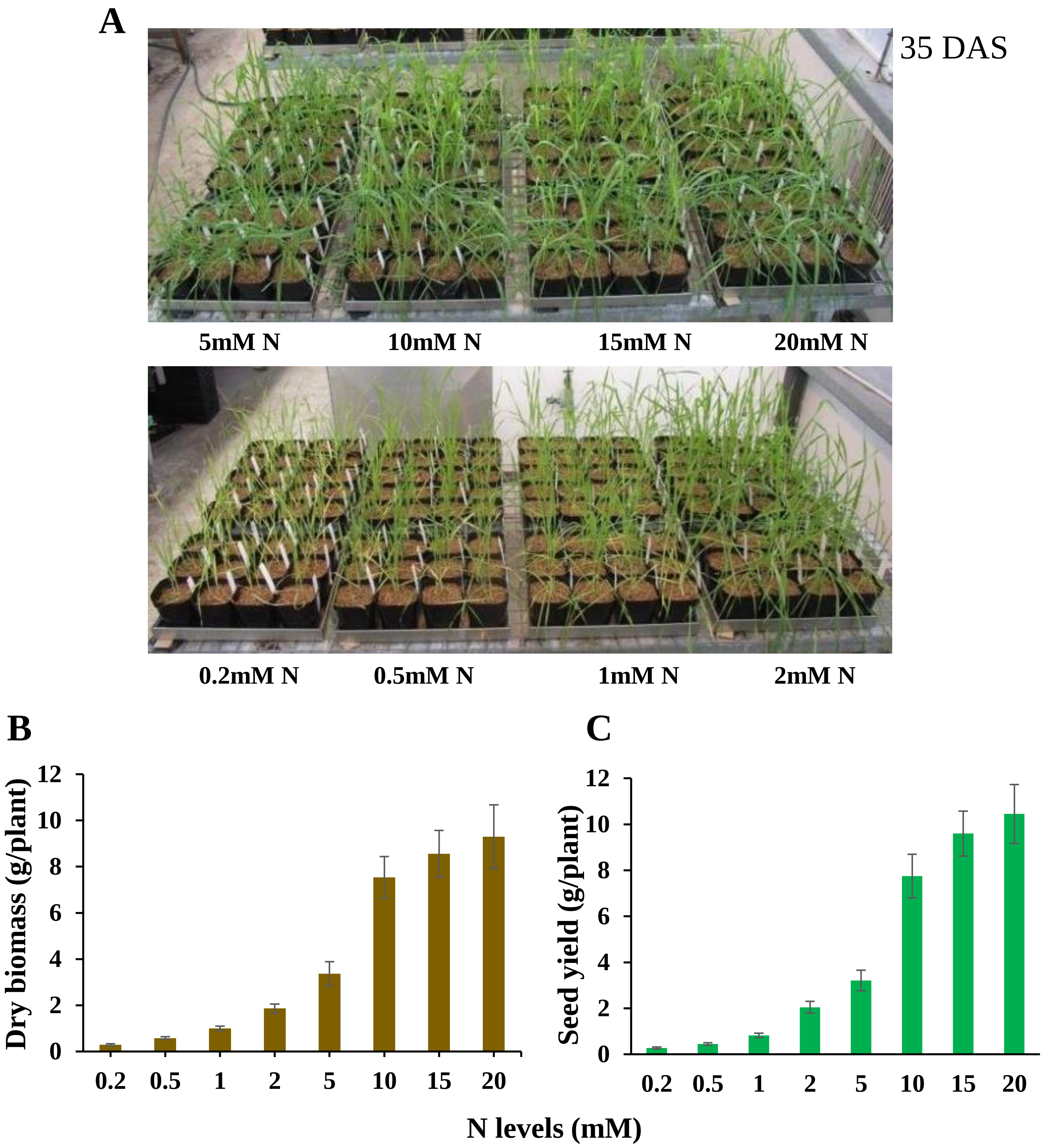

Fig. S1. Growth of wheat plants at different nitrogen levels. (A) Plants of wheat variety Yitpi growing in a normal glasshouse at eight different N levels at 35 DAS. (B) Dry biomass at maturity. (C) Seed yield. Data represent Mean ± SEM. DAS, days after sowing.

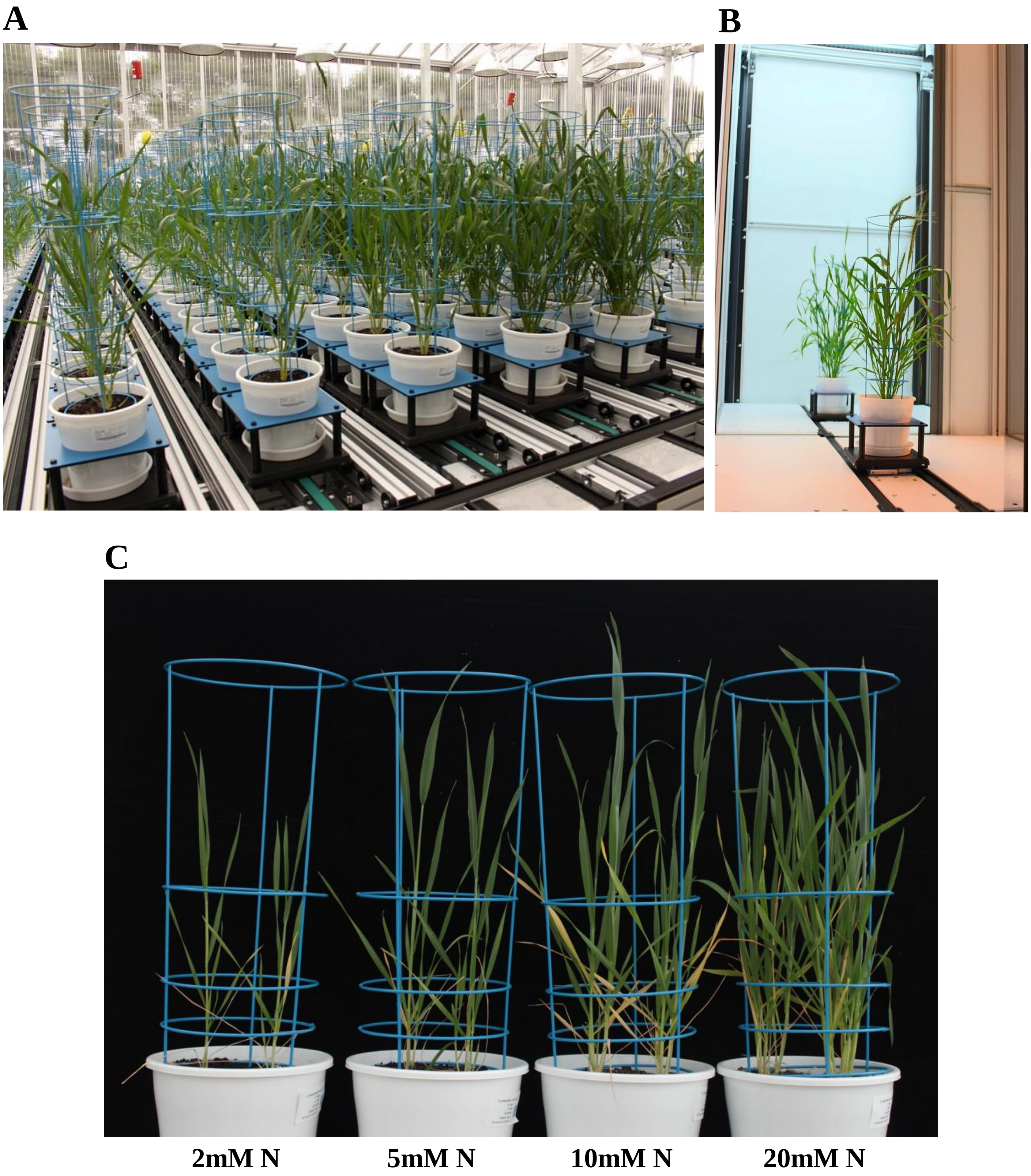

Fig. S2. Wheat plants growing at the Plant Phenomics Victoria, Horsham, automated glasshouse. (A) Plants growing in white plastic pots in carriers on conveyor system. (B) Plants moving through imaging chambers. (C) Growth of wheat variety Yitpi at different nitrogen levels.

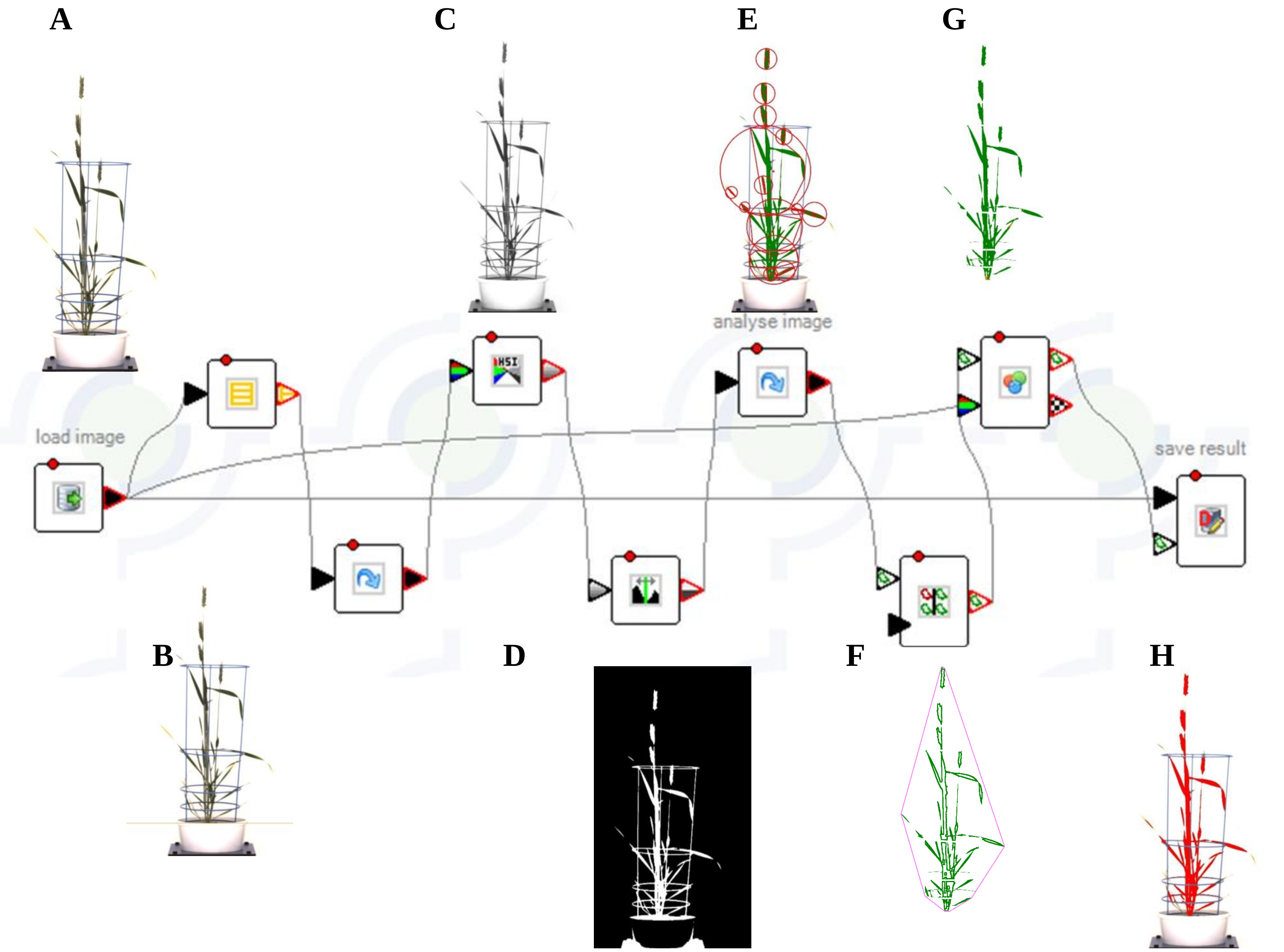

Fig. S3. Simplified RGB colour image analysis pipeline. The key steps and their intermediary output images are shown, (A) input image; (B) selection of region of interest; (C) hue transformation of the image; (D) thresholding; (E) identification of multiple objects; (F) merging of all images into one object as image object composition; (G) colour classification; and (H) plant detection.

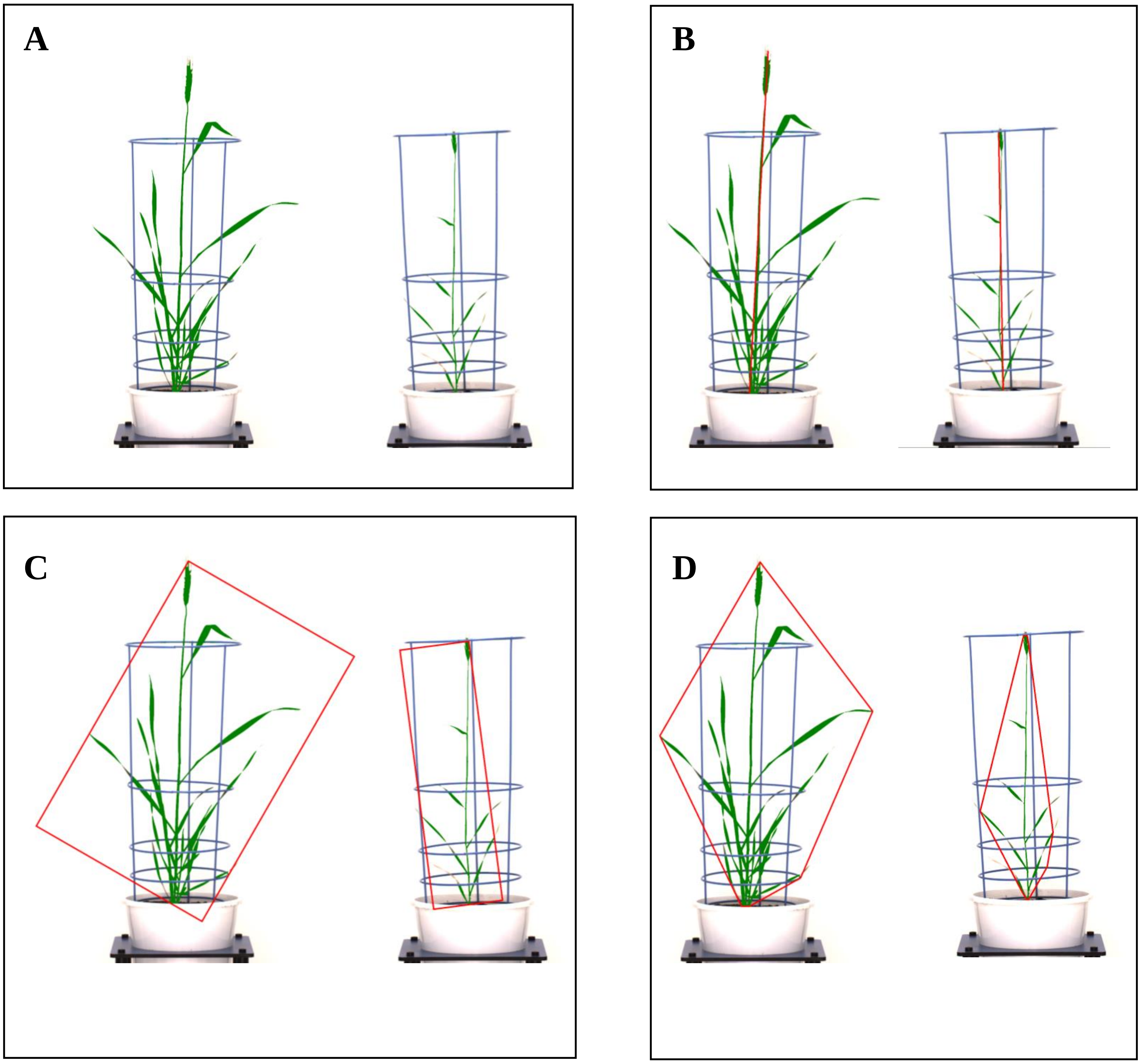

Fig. S4. Estimation of plant digital parameters. (A) detection of whole plant, (B) calliper length, (C) minimum area rectangle, (D) convex hull area. Left panel in each image is plant grown at 20 mM and right panel is plant grown at 5 mM nitrogen.

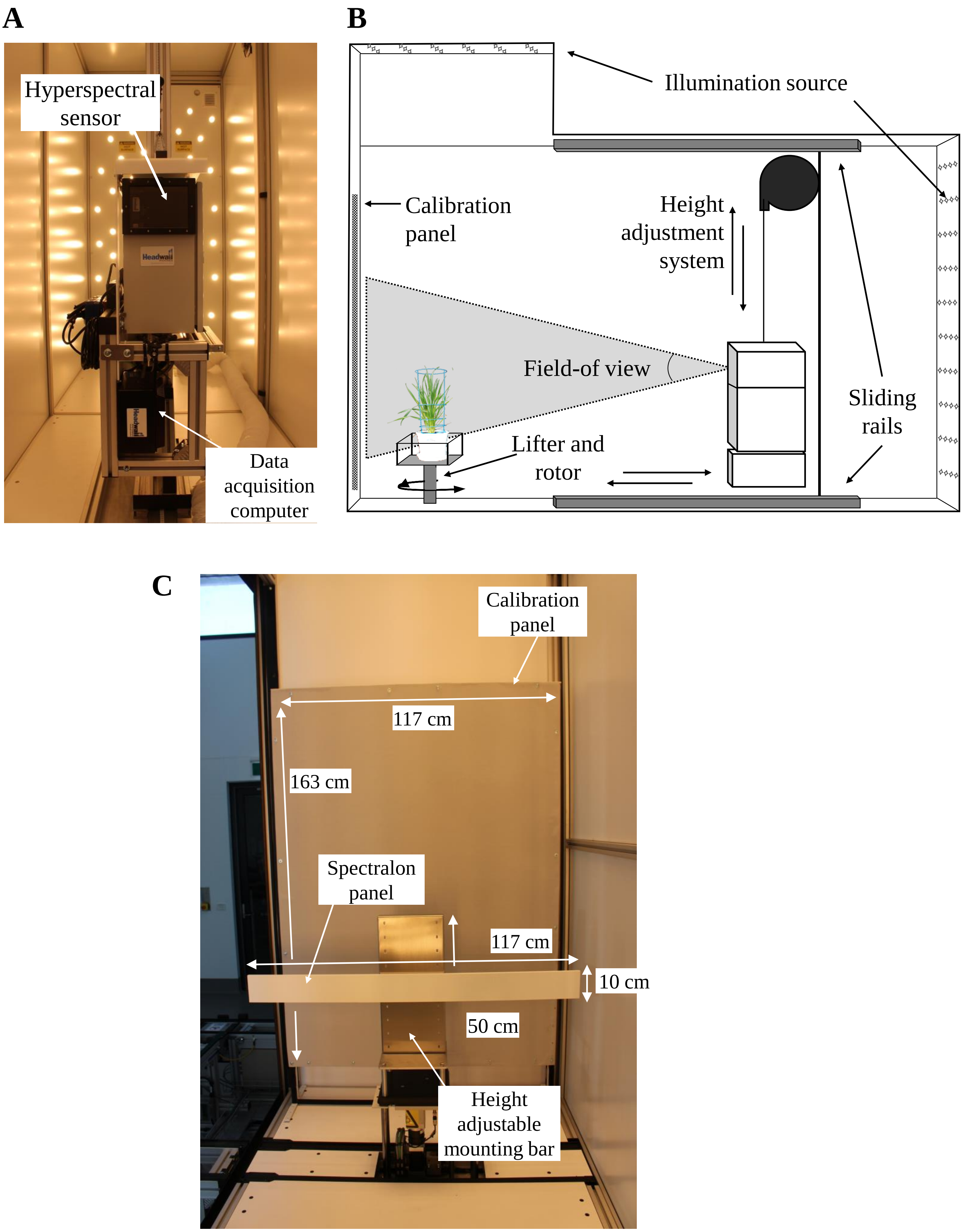

Fig. S5. Hyperspectral sensor system assembly at the Plant Phenomics Victoria, Horsham. (A) Front view. (B) Side view with line diagram illustrating key features. (C) Spectralon panel and persistent background calibration panel layout within imaging cabinet.

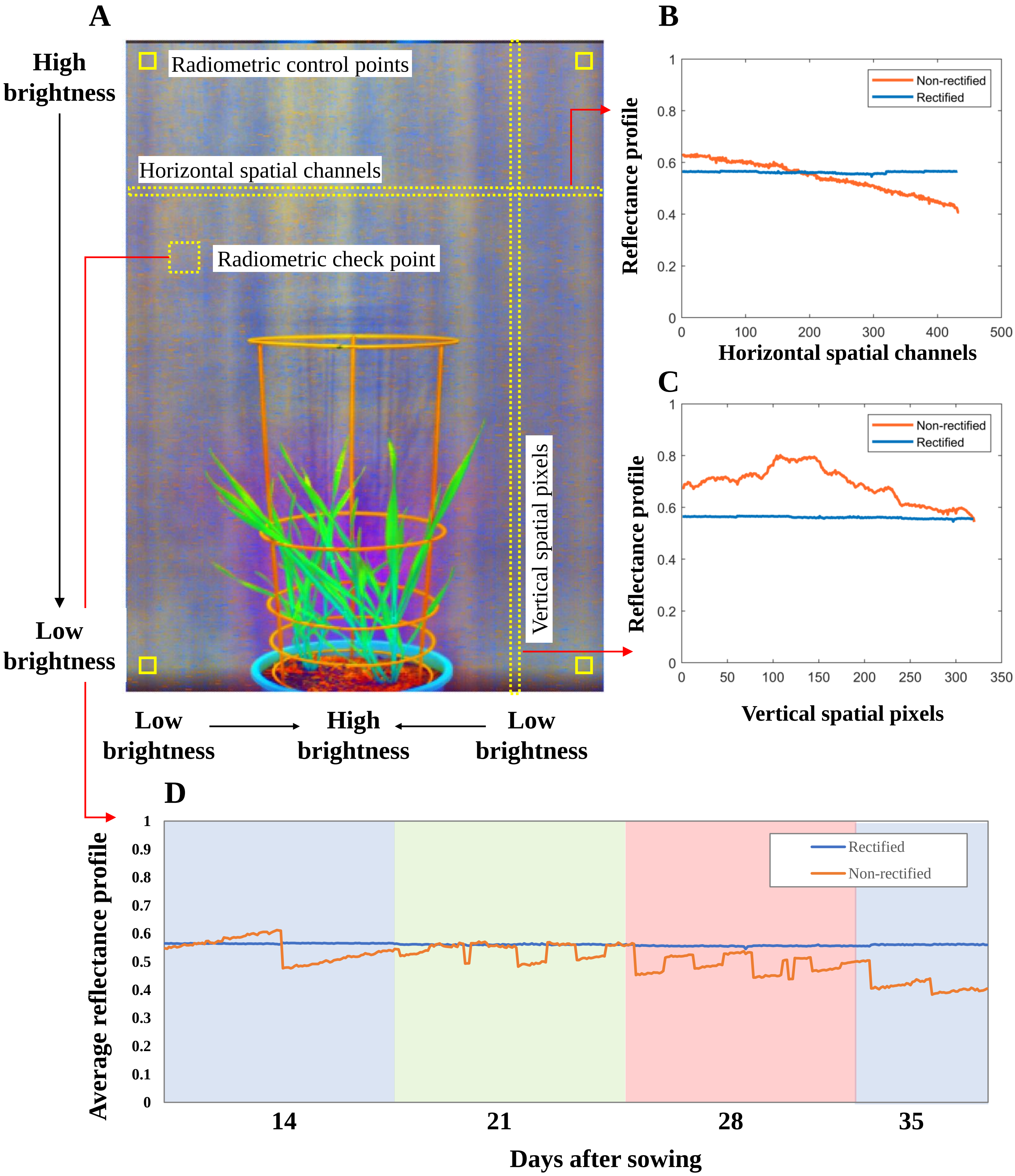

Fig. S6. Calibration of illumination variation in hyperspectral sensing. (A) Radiometrically false color composite image (using 614.91, 1051.95, and 1440.43 nm) from hypercube with identified sampling areas. (B) Horizontal and (C) vertical direction variation rectification in reflectance profile. (D) Variation rectification in average reflectance profile throughout the experiment phase.

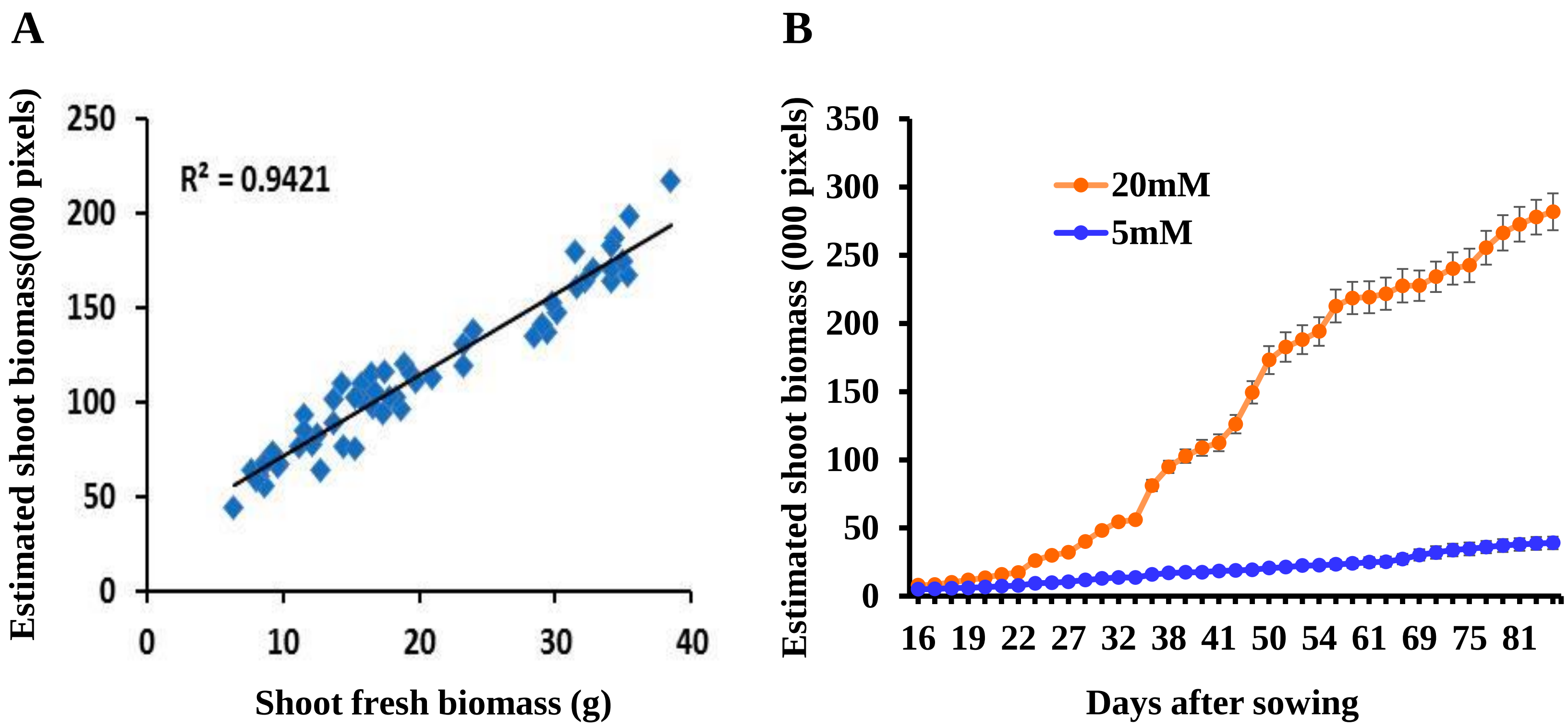

Fig. S7. Estimation of shoot biomass and growth rate of wheat plants. (A) Correlation of estimated shoot biomass calculated after image analysis, and manually harvested shoot fresh biomass. (B) Estimated shoot biomass represented as growth rate of wheat plants grown at two nitrogen levels 5 mM and 20 mM. Data represent Mean ± SEM.

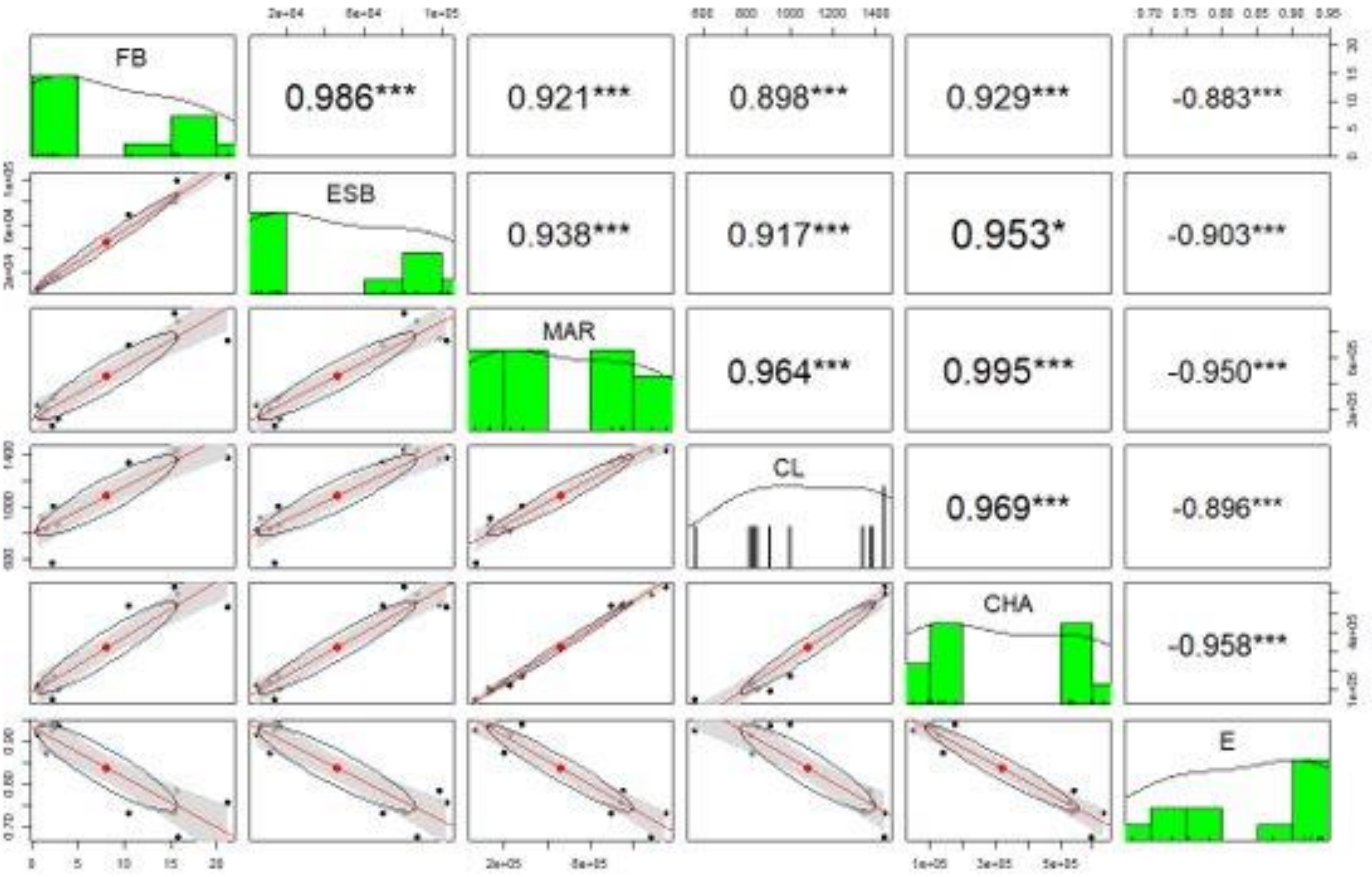

Fig. S8. Correlations between estimated and measured plant parameters. The green histograms and density plots show distribution of the data for each trait; upper right elements show pairwise correlation coefficients (R); the asterisks show the level of significance (*p < 0.05 and ***p < 0.001); lower left elements show pairwise scatter plots with confidence interval; the ellipse in each plot indicate graphical representation of the extent of relationships; the red dot in the middle of ellipse indicates the focus of the ellipse; the red line is the line of best fit resulting from linear regression analysis. FB, fresh biomass; ESB, estimated shoot biomass; MRA, minimum rectangle area; CL, calliper length; CHA, convex hull area; E, eccentricity.

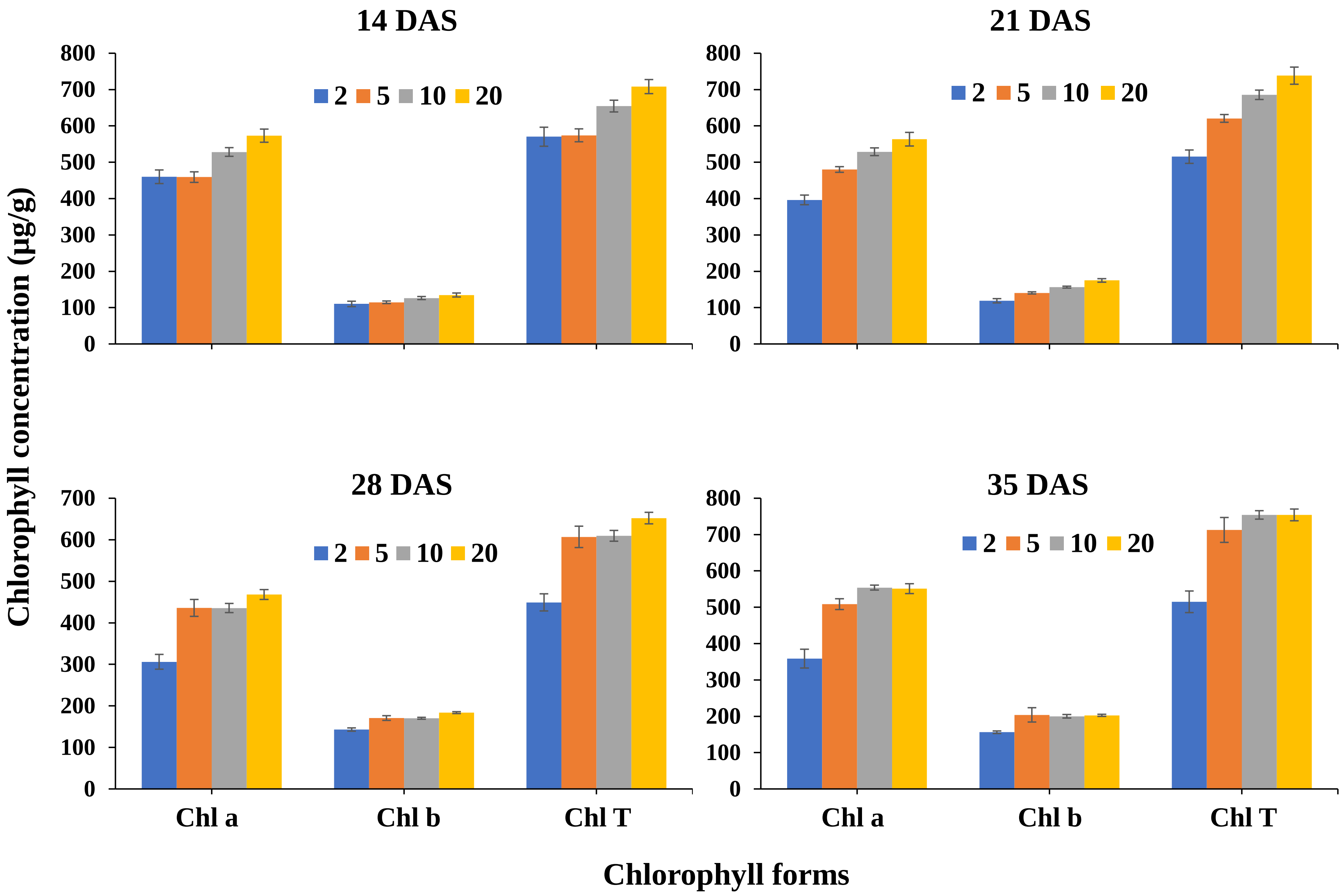

Fig. S9. Chlorophyll analysis of wheat plants. Analysis was done at four time points (14, 21, 28 and 35 DAS), from plants grown at 2 mM, 5 mM, 10 mM and 20 mM nitrogen. Data represent Mean ± SEM. Chl a, chlorophyll a; Chl b, chlorophyll b; Chl T, total chlorophyll. DAS, days after sowing.
