# Supplementary material for: High-throughput phenotyping using digital and hyperspectral imaging-derived biomarkers for genotypic nitrogen response
Source: J Exp Bot. 2020 Mar 18;71(15):4604–15. doi: 10.1093/jxb/eraa143 (PMC7382386; doi:10.1093/jxb/eraa143)
Supplement: eraa143_suppl_Supplementary_Table [file eraa143_suppl_supplementary_table.pdf]

Table S1. Measured and estimated plant traits using destructive harvesting and digital imaging methods.

Plant trait	Description
Fresh Biomass (FB)	The above ground shoot biomass including stem and leaves expressed in grams
Estimated Shoot Biomass (ESB)	Estimated number of pixels from the identified plant involving three side views and one top view expressed in kilo pixels
Minimum Area Rectangle (MAR)	Minimum area that encloses the identified plant expressed in pixels
Calliper Length (CL)	Maximum distance from the bottom to the top of the identified plant expressed as pixels
Convex Hull Area (CHA)	Area of the convex hull that contains the plant expressed in pixels
Eccentricity (E)	Extent of elliptical nature of the plant's spread, generally the value is between 0 and 1

Table S2. Computation of published vegetation indices.

Index	Formula	Reference
Normalized Difference Vegetation Index	$NDVI = \rho800 - \rho680 / \rho800 + \rho680$	Rouse *et al.* (1974)
Simple Ratio Index	$SR = \rho800 / \rho680$	Rouse *et al.* (1974)
Red Edge Normalized Difference Vegetation Index	$RENDVI = \rho750 - \rho705 / \rho750 + \rho705$	Gitelson and Merzlyak (1994)
Vogelmann Red Edge Index 1	$VOG1 = \rho740 / \rho720$	Vogelmann *et al.* (1993)
Vogelmann Red Edge Index 2	$VOG2 = \rho731 - \rho747 / \rho715 + 726$	Vogelmann *et al.* (1993)
Vogelmann Red Edge Index 3	$VOG3 = \rho731 - \rho747 / \rho715 + 720$	Vogelmann *et al.* (1993)
Browning Reflectance Index	$BRI = [(1/ \rho550) - (1/ \rho700)]/ \rho800$	Chivkunova *et al.* (2001)
Modified Chlorophyll Absorption Reflectance Index	$MCARI = ((\rho700 - \rho670) - 0.2(\rho700 - \rho550)) \times \rho700/ \rho670$	Daughtry *et al.* (2000)
Modified Chlorophyll Absorption Reflectance Index 2	$MCARI2 = 1.5 \times [2.5 \times (\rho800 - \rho670) - 1.3(\rho800 - \rho550)] \div \sqrt{((2 \times \rho800 + 1)2) - (6 \times \rho800 - 5 \times \sqrt{}\rho670) - 0.5}$	Haboudane *et al.* (2004)
Modified Triangular Vegetation Index	$MTVI = \rho_{800}1.2 \times [1.2 \times (\rho800 - \rho550) - 2.5 (\rho670 - \rho750)]$	Haboudane *et al.* (2004)
Modified Triangular Vegetation Index 2	$MTVI2 = 1.5 \times [1.2 \times (\rho800 - \rho550) - 2.5(\rho670 - \rho550)] \div \sqrt{((2 \times \rho800 + 1)2) - (6 \times \rho800 - 5 \times \sqrt{}\rho670) - 0.5}$	Haboudane *et al.* (2004)
Transformed Chlorophyll Absorption Reflectance Index	$TCARI = 3 \times [((\rho700 - \rho670) - 0.2(\rho700 - \rho550)) \times \rho700/ \rho670]$	Haboudane *et al.* (2004)
Triangular Vegetation Index	$TVI = 0.5 \times [120 \times (\rho750 - \rho550) - 200 (\rho670 - \rho550)$	Broge and Leblanc (2001)
Anthocyanin Reflectance Index 1	$ARI1 = (1/ \rho550) - (1/ \rho700)$	Gitelson *et al.* (2001)
Anthocyanin Reflectance Index 2	$ARI2 = \rho800[(1/ \rho550) - (1/ \rho700)]$	Gitelson *et al.* (2001)
Water Band Index	$WBI = \rho900/ \rho970$	Gitelson *et al.* (2001)
Normalized Difference Water Index	$NDWI = (\rho857 - \rho1241) / (\rho857 + \rho1241)$	Gao (1996)

Index	Formula	Reference
Moisture Stress Index	$MSI = \rho1599 / \rho819$	Hunt and Rock (1989)
Normalized Difference Infrared Index	$NDII = (\rho819 - \rho1649) / (819 + \rho1649)$	Hardisky *et al.* (1983)
Cercospora Leaf Spot Index	$CLSI = [(\rho698 - \rho570) / (\rho698 + \rho570)] - \rho764$	Mahlein *et al.* (2013)
Modified Chlorophyll Absorpiton Integral	$MCAI = ((\rho545 - \rho752)/2) \times (752 - 545)(\sum_{i=545}^{752} \rho i \times 1.423)$	Laudien *et al.* (2003)
Optimized Soil Adjusted Vegetation Index	$OSAVI = ((1 + 0.16) \times (\rho790 - \rho670))/ (\rho790 - \rho670 + 0.16)$	Rondeaux *et al.* (1996)
Global Environmental Monitoring Index	$GEMI = eta \times (1 - 0.25.eta) - (\rho680 - 0.125/1 - \rho680)$<br>where eta= $(2(\rho^2 800- \rho^2 680) +1.5* \rho800+0.5 * \rho680)/ \rho800+ \rho680+0.5$	Pinty and Verstraete (1992)
Difference Vegetation Index	$DVI = \rho800 - \rho680$	Tucker (1979)
Green Difference Vegetation Index	$GDVI = \rho800 - \rho550$	Sripada *et al.* (2006)
Green Normalized Difference Vegetation Index	$GNDVI = (\rho800 - \rho550) / (\rho\, 800 + \rho550)$	Gitelson and Merzlyak (1998)
Green Ratio Vegetation Index	$GRVI = \rho800/ \rho550$	Sripada *et al.* (2006)
Infrared Percentage Vegetation Index	$IPVI = \rho800 / (\rho\, 800 + \rho680)$	Crippen (1990)
Renormalized Difference Vegetation Index	$RDVI = (\rho800 - \rho680)/ \sqrt{\rho800 + \rho680}$	Roujean and Breon (1995)
Modified Simple Ratio	$MSR = [(\rho800/ \rho800) - 1] / \sqrt{\rho800/ \rho800) + 1}$	Chen (1996)
Non-Linear Index	$NLI = (\rho8002 - \rho680)/ (\rho8002 + \rho680)$	Goel and Qin (1994)
Modified Non-Linear Index	$MNLI = (\rho8002 - \rho680) \times (1 + L) / (\rho8002 + \rho680 + L)$<br>Where L=0.5	Peng *et al.* (2003)
Soil Adjusted Vegetation Index	$SAVI = [1.5 \times (\rho800 - \rho680)]/ (\rho800 + \rho680 + 0.5)$	Huete (1988)
Transformed Difference Vegetation Index	$TDVI = \sqrt{0.5 + [(\rho800 - \rho680)/(\rho800 + \rho680)]}$	Bannari *et al.* (2002)
Red Edge Position Index	$REP = arg\, max\, wl\, (\rho690 - 740)$	Baranoski and Rokne (2005)
Gitelson and Merzlak Index 1	$GMI1 = \rho750/ \rho550$	Gitelson and Merzlyak (1998)
Gitelson and Merzlak Index 2	$GMI2 = \rho750/ \rho700$	Gitelson and Merzlyak (1998)
Greenness Index	$G = \rho554/ \rho677$	Smith *et al.* (1995)

Index	Formula	Reference
Lichtenthaler Index 1	$LIC1 = (\rho 790 - \rho 680)/(\rho 790 + \rho 680)$	Lichtenthaler (1996)
Nitrogen Related Index NRI1510	$NRI1510 = (\rho 1510 - \rho 660)/(\rho 1510 + \rho 660)$	Herrmann *et al.* (2010)
Nitrogen Related Index NRI850	$NRI850 = (\rho 850 - \rho 660)/(\rho 850 + \rho 660)$	Behrens *et al.* (2006)
Water Index	$WI = \rho 1450/\rho 1050$	Thiel *et al.* (2010)
Disease Water Stress Index 4	$DWSI4 = \rho 550/\rho 680$	Apan *et al.* (2003)
Carter Index 2	$CRT2 = \rho 695/\rho 760$	Carter (1994)
Zarco-Tejada & Miller Index (ZMI) or Chlorophyll Index (CI)	$ZMI = \rho 750/\rho 710$	Zarco-Tejada *et al.* (2001)
Leaf Structure Index	$LSI = \rho 1110/\rho 810$	Sridhar *et al.* (2007)
Photochemical Reflectance Index	$PRI = (\rho 531 - \rho 570)/(\rho 631 + \rho 570)$	Gamon *et al.* (1997)
Normalized Difference Chlorophyll Index	$NDCIw = (\rho 727 - \rho 1654)/(\rho 727 + \rho 1654)$	In this paper

References

Apan A, Held A, Phinn S, Markley J. 2003. Formulation and assessment of narrow-band vegetation indices from EO1 Hyperion imagery for discriminating sugarcane disease. *Spatial Sciences Institute Biennial Conference (SSC 2003): Spatial Knowledge Without Boundaries*. Canberra, Australia, 1-13.

Bannari A, Asalhi H, Teillet P. 2002. Transformed difference vegetation index (TDVI) for vegetation cover mapping. *IEEE International Geoscience and Remote Sensing Symposium*, Vol. 5, 3053-3055.

Baranoski GVG, Rokne JG. 2005. A practical approach for estimating the red edge position of plant leaf reflectance. International Journal of Remote Sensing **26**, 503-521.

Behrens T, Müller J, Diepenbrock W. 2006. Utilization of canopy reflectance to predict properties of oilseed rape (*Brassica napus* L.) and barley (*Hordeum vulgar*e L.) during ontogenesis. European Journal of Agronomy **25**, 345-355.

Broge NH, Leblanc E. 2001. Comparing prediction power and stability of broadband and hyperspectral vegetation indices for estimation of green leaf area index and canopy chlorophyll density. Remote Sensing of Environment **76**, 156-172.

Carter GA. 1994. Ratios of leaf reflectances in narrow wavebands as indicators of plant stress. International Journal of Remote Sensing **15**, 697-703.

Chen JM. 1996. Evaluation of Vegetation Indices and a Modified Simple Ratio for Boreal Applications. Canadian Journal of Remote Sensing **22**, 229-242.

Chivkunova O, Solovchenko A, Sokolova S, Merzlyak M, Reshetnikova I, AA G. 2001. Reflectance Spectral Features and Detection of Superficial Scald–induced Browning in Storing Apple Fruit. Journal of Russian Phytopathological Society **2**, 73-77.

Crippen RE. 1990. Calculating the vegetation index faster. Remote Sensing of Environment **34**, 71-73.

Daughtry CST, Walthall CL, Kim MS, de Colstoun EB, McMurtrey JE. 2000. Estimating Corn Leaf Chlorophyll Concentration from Leaf and Canopy Reflectance. Remote Sensing of Environment **74**, 229-239.

Gamon JA, Serrano L, Surfus JS. 1997. The Photochemical Reflectance Index: An Optical Indicator of Photosynthetic Radiation Use Efficiency across Species, Functional Types, and Nutrient Levels. Oecologia **112**, 492-501.

Gao B-c. 1996. NDWI - A normalized difference water index for remote sensing of vegetation liquid water from space. Remote Sensing of Environment **58**, 257-266.

Gitelson A, Merzlyak MN. 1994. Spectral Reflectance Changes Associated with Autumn Senescence of *Aesculus hippocastanum* L. and *Acer platanoides* L. Leaves. Spectral Features and Relation to Chlorophyll Estimation. Journal of Plant Physiology **143**, 286-292.

Gitelson AA, Merzlyak MN. 1998. Remote sensing of chlorophyll concentration in higher plant leaves. Advances in Space Research **22**, 689-692.

Gitelson AA, Merzlyak MN, Chivkunova OB. 2001. Optical Properties and Nondestructive Estimation of Anthocyanin Content in Plant Leaves. Photochemistry and Photobiology **74**, 38-45.

Goel NS, Qin W. 1994. Influences of canopy architecture on relationships between various vegetation indices and LAI and Fpar: A computer simulation. Remote Sensing Reviews **10**, 309-347.

Haboudane D, Miller J, Pattey E, PJ Z-T, Strachan I. 2004. Hyperspectral vegetation indices and novel algorithms for predicting green LAI of crop canopies: Modeling and validation in thecontext of precision agriculture. Remote Sensing of Environment **90**, 337-352.

Hardisky M, Klemas V, Smart R. 1983. The influence of soil salinity, growth form, and leaf moisture on the spectral radiance of *Spartina alterniflora* canopies. Photogrammetric Engineering and Remote Sensing **49**, 77-83.

Herrmann I, Karnieli A, Bonfil DJ, Cohen Y, Alchanatis V. 2010. SWIR-based spectral indices for assessing nitrogen content in potato fields. International Journal of Remote Sensing **31**, 5127-5143.

Huete AR. 1988. A soil-adjusted vegetation index (SAVI). Remote Sensing of Environment **25**, 295-309.

Hunt ER, Rock BN. 1989. Detection of changes in leaf water content using Near- and Middle-Infrared reflectances. Remote Sensing of Environment **30**, 43-54.

Laudien R, Bareth G, Doluschitz R. 2003. Analysis of hyperspectral field data for detection of sugar beet diseases. *Proceedings of the EFITA conference*, Vol. 59. Debrecen, Hungary, 375-381.

Lichtenthaler HK. 1996. Vegetation Stress: An Introduction to the Stress Concept in Plants. Journal of Plant Physiology **148**, 4-14.

Mahlein AK, Rumpf T, Welke P, Dehne HW, Plümer L, Steiner U, Oerke EC. 2013. Development of spectral indices for detecting and identifying plant diseases. Remote Sensing of Environment **128**, 21-30.

Peng G, Ruiliang P, Biging GS, Larrieu MR. 2003. Estimation of forest leaf area index using vegetation indices derived from Hyperion hyperspectral data. IEEE Transactions on Geoscience and Remote Sensing **41**, 1355-1362.

Pinty B, Verstraete M. 1992. GEMI: a non-linear index to monitor global vegetation from satellites Vegetatio **101**, 15-20.

Rondeaux G, Steven M, Baret F. 1996. Optimization of soil-adjusted vegetation indices. Remote Sensing of Environment **55**, 95-107.

Roujean J-L, Breon F-M. 1995. Estimating PAR absorbed by vegetation from bidirectional reflectance measurements. Remote Sensing of Environment **51**, 375-384.

Rouse J, Haas R, Schell J, Deering D. 1974. Monitoring vegetaion systems in the Great Plains with ERTS. In: Fraden SC, Marcanti EP, Becker MA, eds. *NASA. Goddard Space Flight Center 3d ERTS-1 Symposium*, Vol. 1. Texas, USA: NASA, 309-317.

Smith R, Adams J, Stephens D, Hick P. 1995. Forecasting wheat yield in a Mediterranean-type environment from the NOAA satellite. Australian Journal of Agricultural Research **46**, 113-125.

Sridhar BBM, Han FX, Diehl SV, Monts DL, Su Y. 2007. Spectral reflectance and leaf internal structure changes of barley plants due to phytoextraction of zinc and cadmium. International Journal of Remote Sensing **28**, 1041-1054.

Sripada RP, Heiniger RW, White JG, Meijer AD. 2006. Aerial Color Infrared Photography for Determining Early In-Season Nitrogen Requirements in Corn Agronomy Journal **98**, 968-977.

Thiel M, Tath T, Ruckelshausen A. 2010. Plant moisture measurement in field trials based on NIR spectral imaging - a feasibility study. *CIGR Workshop on Image Analysis in Agriculture*. Budapest, Hungary, 16-29.

Tucker CJ. 1979. Red and photographic infrared linear combinations for monitoring vegetation. Remote Sensing of Environment **8**, 127-150.

Vogelmann JE, Rock BN, Moss DM. 1993. Red edge spectral measurements from sugar maple leaves. International Journal of Remote Sensing **14**, 1563-1575.

Zarco-Tejada P, JR M, Noland T, Mohammed G, Sampson P. 2001. Scaling-up and model inversion methods with narrow-bandoptical indices for chlorophyll content estimation in closed forest canopies with hyperspectral data. IEEE Transactions on Geoscience and Remote Sensing **39**, 1491-1507.

Table S3. Correlation analysis of standard vegetation indices and $NDCI_w$ for chlorophyll (Chl) estimation.

Vegetation indices	R^2 for Experiment 1			R^2 for Experiment 2		
	**Chl a**	**Chl b**	**Total Chl**	**Chl a**	**Chl b**	**Total Chl**
NDVI	0.474295	0.509662	0.487518	0.491791	0.135348	0.410487
SR	0.488232	0.538561	0.505281	0.515934	0.169509	0.440774
RENDVI	0.521022	0.554114	0.534116	0.738094	0.347815	0.665033
VOG1	0.648151	0.690965	0.664850	0.806719	0.484770	0.757069
VOG2	0.733895	0.795271	0.756003	0.794843	0.484678	0.747854
VOG3	0.732590	0.795708	0.755116	0.792421	0.480510	0.744839
BRI	0.225153	0.194883	0.219289	0.320426	0.043175	0.247544
MCARI	0.038788	0.049697	0.041793	0.648110	0.762985	0.696829
MCARI2	0.593263	0.609017	0.602676	0.542270	0.169423	0.460139
MTVI	0.617325	0.648715	0.630888	0.578882	0.213315	0.502553
MTVI2	0.593263	0.609017	0.602676	0.542270	0.169423	0.460139
TCARI	0.318387	0.228395	0.296946	0.901574	0.730975	0.894904
TVI	0.544092	0.570477	0.555725	0.464264	0.127951	0.387579
ARI1	0.488452	0.496980	0.495080	0.539050	0.176886	0.460445
ARI2	0.584794	0.629752	0.601432	0.601502	0.218934	0.521270
WBI	0.173482	0.230246	0.188766	0.541919	0.233605	0.481530
NDWI	0.166353	0.227115	0.182455	0.508988	0.210801	0.449527
MSI	0.173482	0.230246	0.188766	0.541919	0.233605	0.481530
NDII	0.283460	0.335835	0.298972	0.561284	0.244380	0.499503
CLSI	0.222335	0.249116	0.231045	0.011723	0.191891	0.033416
MCAI	0.055895	0.071141	0.060116	0.663695	0.339198	0.605906
OSVI	0.487154	0.525872	0.501328	0.601607	0.221852	0.522336
GEMI	0.487154	0.525872	0.501328	0.601607	0.221852	0.522336
DVI	0.507273	0.548149	0.522171	0.555108	0.218886	0.486683
GDVI	0.312827	0.360210	0.327439	0.725026	0.410506	0.673397
GNDVI	0.335762	0.387125	0.351568	0.749775	0.437823	0.700109
GRVI	0.344370	0.399007	0.361054	0.742656	0.430059	0.692456
IPVI	0.474295	0.509662	0.487518	0.491791	0.135348	0.410487
RDVI	0.501915	0.542236	0.516625	0.548863	0.190213	0.472372
MSR	0.482170	0.525238	0.497372	0.510522	0.163555	0.434661
NLI	0.488306	0.531192	0.503522	0.572074	0.235356	0.504738
MNLI	0.494839	0.541685	0.511094	0.589746	0.253395	0.523767
SAVI	0.479150	0.517258	0.493098	0.524520	0.169455	0.447085
TDVI	0.469552	0.500606	0.481659	0.487016	0.131663	0.405589
REP	0.706759	0.740519	0.721742	0.716657	0.457175	0.658663
GMI1	0.207687	0.250321	0.220068	0.718771	0.358920	0.653697
GMI2	0.481757	0.525681	0.497167	0.556157	0.209267	0.484279
G	0.511464	0.511268	0.516086	0.099128	0.004815	0.055714

LIC1	0.473264	0.508249	0.486383	0.491833	0.136749	0.411053
NRI1510	0.774672	0.752410	0.776045	0.363436	0.214101	0.339883
NRI1850	0.492067	0.532713	0.506765	0.667474	0.284055	0.591931
WI	0.273569	0.324587	0.288652	0.644882	0.285833	0.575483
DWSI4	0.501752	0.498760	0.505572	0.123402	0.001894	0.073341
CRT2	0.446858	0.473287	0.457602	0.453804	0.117735	0.376002
ZMI	0.593798	0.644070	0.611837	0.792200	0.439726	0.733294
LSI	0.004824	0.003051	0.004378	0.336525	0.082091	0.276750
PRI	0.580612	0.564471	0.581781	0.760401	0.365712	0.687369
$NDCI_w$	0.930600	0.925800	0.937800	0.912998	0.805845	0.921944
